# Behavioral and Emotional Disorders and Transportation Noise among Children and Adolescents: A Systematic Review and Meta-Analysis

**DOI:** 10.3390/ijerph16183336

**Published:** 2019-09-10

**Authors:** Melanie Schubert, Janice Hegewald, Alice Freiberg, Karla Romero Starke, Franziska Augustin, Steffi G. Riedel-Heller, Hajo Zeeb, Andreas Seidler

**Affiliations:** 1 Institute and Policlinic of Occupational and Social Medicine, Faculty of Medicine, Technische Universität Dresden, 01307 Dresden, Germany; melanie.schubert@tu-dresden.de (M.S.); alice.freiberg@tu-dresden.de (A.F.); karla.romero_starke@tu-dresden.de (K.R.S.); franzi_augustin@web.de (F.A.); andreas.seidler@mailbox.tu-dresden.de (A.S.); 2 Institute of Social Medicine, Occupational Health and Public Health, University of Leipzig, 04103 Leipzig, Germany; steffi.riedel-heller@medizin.uni-leipzig.de; 3 Department of Prevention and Evaluation, Leibniz-Institute for Prevention Research and Epidemiology – BIPS GmbH, 28359 Bremen, Germany; zeeb@bips.uni-bremen.de; 4 Health Sciences Bremen, University of Bremen, 28344 Bremen, Germany

**Keywords:** noise, transportation, traffic noise, noise pollution, road traffic noise, aircraft noise, railway noise, anxiety, depression, disruptive behavior disorders, psychology, cognition disorders

## Abstract

Children and adolescents may be particularly vulnerable to environmental influences such as noise which can affect mental well-being. The aim of this systematic review was to evaluate the effect of transportation noise on behavioral and emotional disorders in children and adolescents using a meta-analytic approach. Therefore, we searched four databases (Pubmed, Embase, PsychINFO, and PSYNDEX) and grey literature until February of 2019. We identified 14 articles from 10 studies examining the effect of transportation noise exposure on the mental health of children. These studies predominately used the *Strength and Difficulties Questionnaire* (SDQ) and mainly focused on schoolchildren and adolescents aged 9–10 years and 15–17 years in Europe. Three studies could be included in the meta-analysis. In sum, the odds for hyperactivity/inattention and total difficulties was significantly increased by 11% (Odds Ratio, OR = 1.11 (95% Confidence Interval, CI 1.04–1.19), respectively 9% (95% CI 1.02–1.16) per 10 dB road traffic noise. Thus, we obtained evidence for an effect of road traffic noise on hyperactivity/inattention and total difficulties, although we could consider few studies. Future studies are needed that use similar techniques to assess outcomes and exposures at schools and in homes. This would make it possible to conduct an individual participant data pooled analysis of the data.

## 1. Introduction

Mental disorders and their consequences impose not only health and social costs but may also lead to the victimization, discrimination and stigmatization of patients [1]. According to the World Health Organization [2], they are “the leading cause of disability and the third leading cause of overall disease burden (measured as disability-adjusted life years)”. Mental health disorders are most prevalent, and nearly 30% of the population experiences a common mental disorder at some time during their life [3].

For children and adolescents, a recent meta-analysis estimated a global prevalence of mental health disorders of 13.5% (95% Confidence Interval, CI 11.3–15.9) [4]. Anxiety disorders were the most prevalent mental disorder (6.5% (95% CI 4.7–9.1)) followed by disruptive disorder (5.7% (95% CI 4.0–8.1)), hyperactivity disorder (3.4% (95% CI 2.6–4.5)), and any depressive disorder (2.6% (95% CI 1.7–3.9)). A prior meta-analysis by the same research group focused solely on attention deficit hyperactivity disorder (ADHD)/hyperkinetic disorder (HD) and found a prevalence of 5.3% (95% CI 5.0–5.6, Polanczyk et al. [5]). An analysis of German Health claims data shows that the ADHD diagnosis prevalence increased from about 2.4% in 2001 to 6.1% in 2014 [6,7,8,9]. ADHD may also persist into adulthood [10]. A WHO survey on adult ADHD showed a prevalence of 3.6% in high income countries. Adult ADHD is significantly associated with role impairment (e.g., impaired cognition, social interactions), and is highly comorbid with anxiety, mood, behavior, and substance disorders [11]. Conduct disorders are also common among children, and are defined as consistent patterns of anti-social behavior presenting in childhood or adolescence [12]. A population-based survey in Great Britain found 5% of children aged 5–15 years had a clinically relevant conduct disorder [13].

According to Stansfeld and Matheson [14], children may be “particularly vulnerable to the non-auditory health effects of noise,” as they are still growing and developing cognitively, and may not be as capable of developing strategies for coping with noise as adults. While the body of evidence regarding the effects of environmental noise exposure on adult health, especially cardiovascular health, is accumulating [15,16], the impact of environmental noise on the health and development in children is less clear. 

Studies indicate that chronic noise exposure impacts children’s wellbeing and annoyance [17,18]. Recently, Bergström and colleagues [19] showed that higher levels of feeling bored at school was associated with a higher residential aircraft noise exposure among German school children. Moreover, children with a higher aircraft noise at home also reported having significantly more head and stomach aches and a worse sleep quality. Another study in Germany found that the odds of sleep problems among children significantly increased with nighttime noise at the least exposed house façade (Odds Ratio, OR = 1.79 (95% CI 1.10–2.92)) [20]. 

Sleep disturbances among children could also impact mental health. It has been shown that children with sleep disorders are often inattentive or hyperactive [21,22,23]. Huhdanpää and colleagues [24] showed that sleep quality and quantity in early childhood (until 24 months) is associated with attention and hyperactivity symptoms at the age of 5 years. An increased risk of attention and hyperactivity problems among children has also been associated with higher transportation noise exposure (e.g., [20,25]). 

Recently, the review by Clark and Paunovic [26] examined the body of evidence regarding noise-related risks for emotional conduct disorders among children for the WHO Environmental Noise Guidelines. The authors evaluated the results of emotional conduct disorders (8 studies: 5 aircrafts, 7 roads, 1 railway) and hyperactivity in children (5 studies: 3 aircrafts, 4 roads, 1 railway) published up to 2015. Also, Zare Sakhvidi et al. [27] updated this review through March 2018 and considered the results from 12 studies regarding neurodevelopmental and mental health problems (5 aircrafts, 7 roads, and 1 railway). Clark and Paunovic [26], as well as Zare Sakhvidi et al. [27], point out the heterogeneous results. Potentially harmful effects were found for the effect of road traffic noise on hyperactivity/inattention and the total *Strength and Difficulties Questionnaire* (SDQ) score. Nevertheless, so far the evidence has not been combined in a meta-analysis. Furthermore, Zare Sakhvidi et al. [27] had broad inclusion and exclusion criteria for studies as they also included studies with e.g., community noise or parental noise annoyance as a proxy, as well as a study assessing life quality and not mental health as such, which may have led to a bias of the results. Moreover, further research has been conducted since these studies were published.

We aimed to summarize the current evidence for the effect of transportation noise and mental health in children up to February 2019, by conducting a systematic review and a meta-analysis. We sought cohort, case-control and cross-sectional studies (Study Design), examining people (Populations) exposed to varying levels of road, railway or aircraft noise (Intervention/Comparison), and the effects of noise on the mental health problems. While the systematic search considered mental health problems among adults and children, this paper focuses on emotional and conduct disorders among children, including hyperactivity/inattention (Outcome).

## 2. Materials and Methods 

### 2.1. Research Question and Study Eligibility

We systematically searched for published evidence to determine if individuals exposed to transportation noise (aircraft, road traffic, and railway noise) have an increased risk of psychological complaints and disease by following the procedures outlined in the protocol for our systematic review on non-auditory health complaints and diseases due to aircraft noise registered a priori on PROSPERO (CRD42013006004). This systematic review diverged from the registered study protocol by also considering exposures to road traffic and railway noise, and focusing on mental health complaints.

According to the population-exposure-outcome (PEO) outline, we designed our systematic search strategy to include studies of adults and children (P), and excluded animal studies or studies on occupational populations (i.e., teachers, airport personnel, or road or railway maintenance workers). Exposure (E) to road traffic, railway and/or commercial aircraft noise should have been assessed objectively with actual measurements or noise models. We excluded studies of military aircraft noise, industrial noise, and neighborhood noise. While the search strategy was designed to find all studies examining mental health (O), this paper focuses on the results of the search pertaining to behavioral and emotional disorders occurring in childhood and adolescence (ICD-10: F90–F98). Studies assessing behavioral and emotional disorders with validated screening instruments, such as the Strengths and Difficulties Questionnaire (SDQ) that can identify undiagnosed problems and sub-clinical levels of mental health disorders, were also included (Table 1).

We included primary studies using ecological, cross-sectional, case-control, and cohort study designs. We did not include reviews, but assessed the reference lists of reviews for further primary studies. Editorials and letters to the editors, as well as any publication with incomplete information on the study methods and results were excluded from the review. We used no geographic or language restrictions. We also included grey literature that did not appear in a peer-reviewed journal (e.g., conference proceedings, research reports), if enough information on the study methods and results were provided for the assessment of the methodological quality. When information was lacking, we attempted to contact the publication authors.

### 2.2. Information Sources and Search 

We searched the electronic literature databases MEDLINE (Pubmed), Embase (Ovid), PsycINFO (ProQuest), and PSYNDEX (EBSCO host) without any time limitations in February of 2019. Pubmed was searched using the search string: (“depression” OR “affective” OR “anxiety” OR “panic” OR "dysthymia" OR “dementia” OR Alzheimer* OR “mental” OR psychi* OR psychol* OR “annoyance”) AND (“noise” AND (“aircraft” OR “airways” OR airplane* OR airline* OR “jet” OR “flight” OR rail* OR “train” OR “road” OR “highway” OR “street” OR “traffic” OR “transport”)). This search string was adapted for the other databases, accordingly.

Reviews [26,27] and references of the included studies were searched for additional references. We also searched the proceedings of the German Society for Acoustic (*Deutsche Gesellschaft für Akustik*, DAGA) and the INTER-NOISE conferences for studies. 

### 2.3. Study Selection and Data Collection 

The search results were imported into an Endnote reference management system database, and duplicate references were removed at import. The titles and abstracts were screened independently by two authors (MS and JH) for inclusion and exclusion criteria. Disagreements regarding the inclusion were discussed and often included in the full-text screening to err on the side of caution. The full-texts of articles were screened by two independent reviewers, and disagreements were resolved in meetings. 

We extracted the following study characteristics:

- study design,
- region,
- study population size,
- population characteristics (age and sex distributions),
- population sampling information (recruitment times, response and follow-up),
- outcomes considering how they were assessed (instruments used),
- noise exposures sources considered,
- noise assessment, including the noise levels considered, and
- relevant study results.

Further details regarding the study, such as the adjustment for confounders, conflicts of interest, and funding sources were also extracted in the comments section of our extraction form (Supplementary Material, Table S1). The extraction of the data was done by one reviewer (FA, KR, or MS) and checked for accuracy by a second reviewer (AF). Either the fully-adjusted results or the results of what the authors described as their “main analyses” were extracted and included in the meta-analysis.

### 2.4. Rating of Methodological Study Quality 

At least two reviewers (KR, FA, AF, JH, AS, and MS) assessed the methodological quality of the studies using a hybrid tool that was used previously for other reviews of noise-related health effects [16] and occupational exposures [28,29]. This hybrid tool combines characteristics of both the SIGN (Scottish Intercollegiate Guidelines Network 2004) and CASP (Critical Appraisal Skills Programme 2004/2006) assessment tools, and considers several domains (e.g., confounding, exposure assessment, selection bias). An example of the checklist was published in [29]. 

A study was given the quality rating ‘++’ if a majority of the quality criteria were met and if it was *very unlikely* that the study results were biased by the unmet criteria. If only some of the quality criteria were met, but it was still *unlikely* that the study results were biased by the unmet criteria, the study was given a rating of ‘+’. A study that met few or none of the quality criteria was given a ‘–’ rating, because the reported results were *likely* or *very likely* due to unresolved bias. The assessments of the methodological study quality also take into account that ecological or cross-sectional studies typically cannot contribute the same level of scientific evidence as prospective cohorts or even case-control studies can. The study quality ratings and the reasoning behind the quality ratings are also included under the comments section of the extraction form (Supplementary Material, Table S2). Divergent assessments of quality were discussed in meetings and resolved collectively.

### 2.5. Meta-Analysis

A meta-analysis was conducted to obtain pooled risk estimates per 10 dB if at least two studies considering the same exposure source and outcome were available. If necessary, the published estimates were converted to risks per 10 dB day-evening-night weighted−24 h means (L_DEN_) by first converting the noise metrics to L_DEN_ using the conversion factors proposed by Brink et al. [30] and then calculating the risks per 10 dB L_DEN_. For example, an estimated OR reported per 20 dB day-night weighted means (L_DN_) of road traffic noise would be converted to an OR per 10 dB L_DEN_ as follows:

(1) $$e^{\left(\frac{\ln\left(OR\right)}{20\;dB\;L_{DN}\;}\;\times \;\frac{1\;dB\;L_{DN}}{1.5\;dB\;L_{DEN}}\;\times 10\;dB\;L_{DEN}\right)\;}$$

The Stata package *metan* was used to conduct the meta-analysis of the traffic noise risk estimates per 10 dB and to create forest plots. We intended to use the random-effects model for the meta-analysis, but because only few studies could be included in the analysis and the between-study variance (τ^2^) may not be correctly estimated with so few studies [31], we decided to use the fixed effect model for the meta-analysis instead.

We planned to conduct a sensitivity analyses by excluding studies with a low methodological quality when at least one study with a ‘+’ or ‘++’ rating was available. This would help to determine the possible direction and to impact a potential study bias on the pooled results. Where five or more studies were available, we planned to visually consider the publication bias using a funnel plot. There were too few studies to conduct this sensitivity analysis or the investigation of the publication bias.

## 3. Results

### 3.1. Study Selection

**Figure 1:**
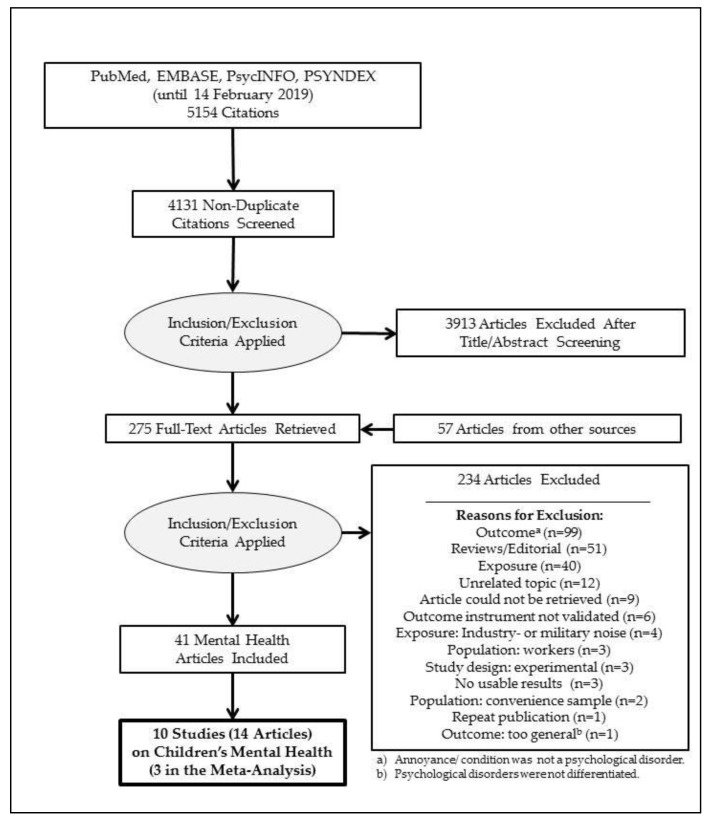
PRISMA flow diagram.

Electronic databases were searched up through February 14, 2019. We screened the full-texts of 218 articles found in the electronic databases and an additional 57 articles found from other sources. We excluded a total of 234 full-text articles from further consideration, and the most common reason for exclusion was that the studies considered annoyance or other complaints that do not correspond to the clinical psychological disorders under consideration (99 studies). Despite extensive efforts by our institute’s librarian, we were unable to locate the full-texts of 9 studies. The references of the non-available full-texts are shown in the supplement. Our reasons for excluding studies are summarized in the PRISMA flow diagram (Figure 1), and the references of the excluded studies and the reasons for exclusion are listed in the Supplementary Material, Table S1. 

### 3.2. Study Characteristics

We identified ten studies published in 14 articles examining the effect of traffic noise exposure on the mental health of children [20,32,33,34,35,36,37,38,39,40,41,42,43,44]. Four of these studies used a longitudinal design [32,34,37,40], while six were cross-sectional studies [20,33,38,39,41,42]. Only the Lim et al. [38] study was conducted in Asia, while the rest were conducted in Europe and the UK.

Seven of the studies assessed behavioral disorders using the validated Strengths and Difficulties Questionnaire (SDQ) [20,32,33,34,37,39,41], which comprises five scales (5 items per scale): (i) emotional symptoms (ii) conduct problems, (iii) hyperactivity/inattention, (iv) peer relationship problems, and (v) prosocial behavior. The scores for the first four scales can be combined to obtain a total difficulties score. Other assessment tools that were used included the Child Behavior Checklist (CBCL) [38] and the Rating Scale for Disruptive Behavior Disorders (RSDBD) [40], which screens for symptoms of attention deficit hyperactivity disorder (ADHD). Belojevic et al. [42] used an adapted version of an Attention Deficit Disorder Questionnaire (5-item-scale). In addition to the SDQ, Haines et al. [34] also screened for depression and anxiety using the Child Depression Inventory (CDI) and the Revised Child Manifest Anxiety Scale (CMAS). Forns et al. [41] also used an Attention Deficit Hyperactivity Disorder – (ADHD)-DSM-IV list that was completed by the teachers. The study characteristics for these studies are shown in Table 2, and the more detailed extraction table is presented in the Supplementary Material, Table S2.

Seven of the studies considered the noise exposure at school, including the four studies conducted by Stephen Stansfeld’s research group that examined the associations between daytime (L_eq, 07–23h_) aircraft and road traffic behavioral disorders among children aged 9–10 years or 15–17 years [32,33,34,39]. Two of the studies looked at the association between transportation noise and psychological outcomes among schoolchildren in the UK [32,34], and two further studies (RANCH-study) also included school children in Spain and the Netherlands [33,39]. Their results indicate that aircraft noise at schools is associated with hyperactivity/inattention, and road noise at schools is associated with conduct problems among school children [33,39]. Haines et al. [34] found no statistically significant increases in depression and anxiety among children exposed to aircraft and road traffic noise. Forns et al. [41] found a significant relationship between a higher ADHD symptomatology according to DSM-IV and higher (traffic-induced) indoor classroom noise levels in Barcelona. Lim et al. [38] also considered noise exposure at schools using a cross-sectional study design. Lim et al. examined the association between the exposure to day-night weighted road traffic noise (L_DN_) and behavioral problems among school children of 4 elementary and secondary schools in Seoul and Ulsan. This study found road traffic noise to be positively associated with internalizing and externalizing problems. A statistically significant increase in the CBCL total score per 1 dB L_DN_ was also observed (OR = 1.08, 95% CI 1.01–1.15). Moreover, Belojevic et al. [42] found a significant association between high noise at home and lower executive functioning in school children from central Belgrade.

Three birth cohorts also considered the impact of early noise exposure at the children’s residence on child development and behavioral problems [20,37,40]. Hjortebjerg et al. [37] considered the effects of road traffic and railway noise (L_night_, L_DEN_) exposures during pregnancy and since birth on the odds of behavioral problems among seven-year olds in the *Danish National Birth Cohort*. Abnormal hyperactivity/inattention was significantly associated with road traffic noise since birth after adjusting for confounders (OR per 10 dB L_DEN_ = 1.10; 95% CI 1.03–1.18). The odds of abnormal hyperactivity/inattention scores due to railway noise exposure at age seven were similar but not statistically significant, while exposure to railway noise at age seven increased the odds of abnormal total difficulties (OR per 10 dB L_DEN_ = 1.13; 95% CI 1.02–1.25) and peer relationship problem scores (OR per 10 dB L_DEN_ = 1.13; 95% CI 1.03–1.25). Road traffic noise exposures during pregnancy and railway traffic noise exposure at birth were not related with later abnormal child behavioral scores. Using data from the *Norwegian Mother and Child Cohort Study,* Weyde et al. [40] also examined associations between exposure to road traffic noise before birth (prenatal), between ages three and eight (five-year average), or road traffic noise exposure at age eight and inattention among eight-year olds. Weyde et al. [40] found that inattention scores increased by 1.2% per 10 dB L_DEN_ at age eight and 1.3% per 10 dB for the five-year average for L_DEN_ (average marginal effects from fractional logit models). 

Tiesler et al. [20] conducted a cross-sectional analysis of residential noise exposure and behavioral problems based on a sample of 10 year olds participating in the German GINI*plus* and LISA*plus* birth cohorts. The relative odds of behavioral problems per increments of the interquartile range for residential road noise at the most exposed façade (8.22 to 9.02 dB L_DEN_) were estimated using logistic regression. Statistically significant odds ratios (OR) for the prevalence of hyperactivity/inattention symptoms were reported (OR = 1.32, 95% CI 1.03–1.58) per interquartile range (IQR) of L_DEN_; OR =1.32, 95% CI 1.06–1.64, per IQR L_eq, 22–6h_. An overview of the study results is shown in Table 3.

#### 3.2.1. Synthesis of Results 

**Figure 2:**
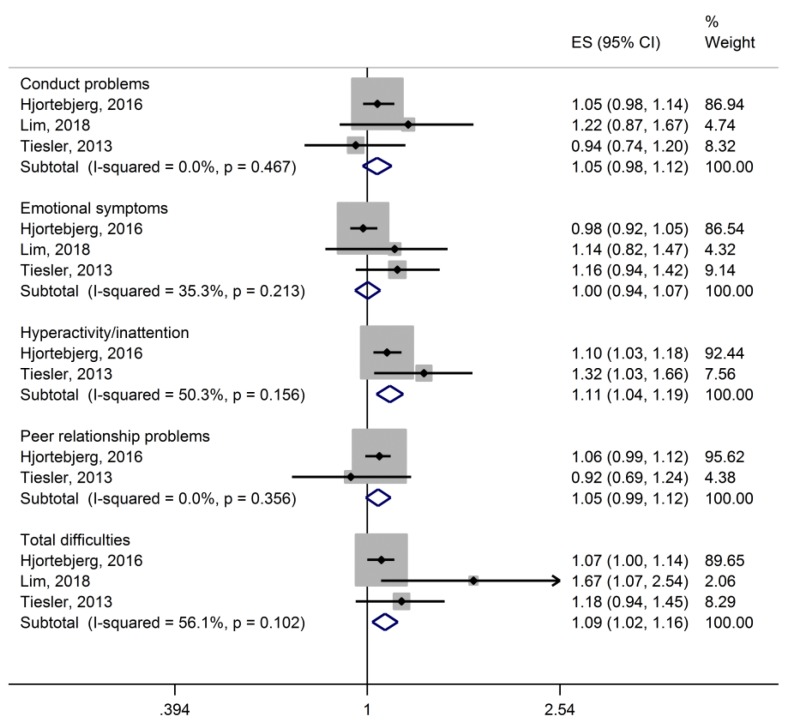
Forest plots of the meta-analysis for the relative risks (effect size, ES) for childhood behavioral problems per 10 dB increase in road traffic noise (L_DEN_). The ESs shown here for Tiesler et al. 2013 (OR per IQR L_DEN_) and Lim et al. 2018 (OR per dB L_DN_) differ from the published results because we converted the effect estimates to represent the risk per 10 dB L_DEN_.

Three studies [20,37,38] reported the risks for abnormal behavioral scores (measured with the SDQ or CBLC) for increases in road traffic noise, and could be included in a meta-analysis (Figure 2). Hjortebjerg et al. [37] provided the risk estimates per 10 dB L_DEN_. Tiesler et al. [20] described the effect sizes per IQR-increase (range 8.22 dB and 9.02 dB for the L_DEN)_. Lim et al. [38] provided the results for a 1 dB increase in L_DN_. The latter two study results were converted to risks per 10 dB L_DEN_.

The results of the meta-analysis shown in Figure 2 indicate a significant association between the road traffic noise, hyperactivity/inattention and total difficulties. In detail, the odds for hyperactivity/inattention was significantly increased by 11% (OR = 1.11; 95% CI 1.04–1.19) and the total difficulties by 9% (OR = 1.09; 95% CI 1.02–1.16) per 10 dB of road traffic noise. We observed an unexplained heterogeneity for the outcomes hyperactivity/inattention, emotional symptoms and total difficulty scores, but not for conduct problems and peer relationship problems. However, this is based on very few effect estimates. 

#### 3.2.2. Risk of Bias Across Studies 

Of the three studies included in the meta-analysis, the studies by Lim et al. [38] and Tiesler et al. [20] were judged to be of a low methodological quality (–), and we only considered the Hjortebjerg et al. [37] study results to be unlikely to be due to bias (+). Hjortebjerg et al. [37] reported only statistically significant increased odds for hyperactivity/inattention and a nearly significant increase for total difficulties. Fewer than five studies could be considered in the meta-analysis, so we did not conduct any analyses of the publication bias or small study effects.

## 4. Discussion

The studies we identified most frequently reported positive associations between noise exposure and hyperactivity/inattention problems. Seven of the nine studies considering hyperactivity and inattention problems as an outcome reported a significant positive association with transportation noise exposure [20,36,37,39,40,41,42], and an eighth study found borderline-significant results [33]. Otherwise, the studies’ results regarding the risks of emotional or behavioral problems among children due to transportation noise exposures were inconclusive. Three studies could be included in the meta-analysis for road traffic noise [20,37,38], and the results showed significantly increased odds for hyperactivity/inattention (OR = 1.11; 95% CI 1.04–1.19) and total difficulties (OR = 1.09; 95% CI 1.02–1.16) per 10 dB L_DEN_.

Regarding the general lack of evidence, although we did not judge the overall quality of the evidence according to the Grading of Recommendations Assessment, Development and Evaluation (GRADE) system, in general our results corroborate the findings of Clark and Paunovic [26]. Clark and Paunovic [26] reported that the current body of evidence for road traffic noise is of moderate quality and indicates that road traffic noise has an effect (the direction was not reported) on conduct disorders among children. They also report that the evidence available for aircraft noise was of low quality and does not indicate any effect. Clark and Paunovic [26] also determined that there was evidence of a harmful effect for railway noise (moderate quality), but that this was based on the results of a single study. We found that since their search in 2015, still only Hjortebjerg et al. [37] considered the effect of railway traffic noise on childhood mental health. In their birth cohort study, Hjortebjerg et al. [37] found an elevated odds ratio (OR = 1.13; 95% CI 1.03–1.25) for peer relationship problems per 10 dB of railway traffic noise, but no increased odds for other behavioral outcomes. 

Zare Sakhvidi et al. [27] also conducted a review of environmental noise exposure and mental health among children, including research published through March 2018. Zare Sakhvidi et al. [27] determined that the overall evidence for the various outcomes considered (e.g., road traffic related or railway traffic related SDQ scores, emotional problems, hyperactivity/inattentions, peer-relation problems, etc.) ranged from low to very low according to their GRADE-system rating. 

Both the Clark and Paunovic [26] and Zare Sakhvidi et al. [27] systematic reviews included studies that we excluded because they did not meet our inclusion criteria. We excluded the Dreger et al. [45] study because noise was not measured or modelled, but instead parental noise annoyance was used as a proxy for noise exposure. We also excluded Ristovska et al. [46] because this study examined community noise, and Lercher et al. [18] because this study considered self-reported well-being, and this did not meet our criteria for (clinical) mental disorders. Despite our more critical inclusion criteria, the findings of our studies are generally in agreement with both reviews. Interestingly, the studies considering noise exposures at schools reported fewer indications of increased mental health risks. However, the results for noise exposure at schools and hyperactivity problems were inconsistent. Five studies considered road and/or aircraft noise exposure at schools and hyperactivity [32,33,36,39,41], of which only Haines et al. [36], [41] and Stansfeld et al. [39] report an association between (aircraft) noise at school and hyperactivity. On the other hand, all four studies of residential noise exposure consistently found an association between (road) traffic noise and inattention/hyperactivity problems [20,37,40,42]. This may indicate that noise exposures at home and during early childhood (two of the residential studies considered noise exposures since birth) may be more relevant to mental health. Moreover, traffic noise at school may also have less impact on mental health because traffic noise levels might be obscured by classroom noise levels. On the other hand, several studies suggest that high traffic noise in schools impairs cognitive development and learning, and increases the annoyance of pupils [47,48,49,50]. It is possible that these noise-related learning problems and annoyance at school are also contributing to the risk of psychological disorders among children.

Both Tiesler et al. [20] and Weyde et al. [40] postulated that noise-related sleep disturbances may be increasing the risks for hyperactivity and inattention problems. Sleep duration has been shown to be associated with psychiatric symptoms among children [51] and hyperactivity [52], based on self-reported cross-sectional studies. While numerous studies report associations between sleep problems or a reduced sleep duration among people with attention-deficit and hyperactivity disorders (ADHD), it is unclear if these are a contributing cause of ADHD or a symptom of the disorder [53].

While Weyde et al. [40] found that transportation noise was not associated with the sleep duration in their cohort, and was therefore not modifying any effect of transportation noise on childhood behavior in this study, the researchers could not determine if transportation noise might otherwise be impacting the sleep quality. Tiesler et al. [20] on the other hand found increased odds of ‘any sleep problems’ with increasing nighttime road traffic noise levels (L_night_) at the least exposed house and no significant association between sleep problems and L_night_ at the most exposed façade. This difference in the estimated risk may indicate that the noise exposures measured at the most exposed house façade are prone to a non-differential misclassification bias, especially if children are likely to have their bedrooms facing less exposed sides.

Although the studies we included in our systematic review, and the studies included in two previous systematic reviews [26,27], differed slightly, all three reviews found that the studies of environmental noise effects on children’s mental health use varied methodologies. This makes it difficult to summarize the findings, and comparisons could be facilitated if future studies used similar exposure and outcome measurement methods. For example, we included studies that measured behavioral problems with either the SDQ or the CBLC. This difference in the outcome assessment could have contributed to the heterogeneity of the risk estimates we observed. However, the scales of these screening instruments and the total (difficulty) scores have been shown to correlate highly [54]. The exposure assessments of the included studies also varied greatly. Tiesler et al. [20] found the most profound effects on children’s behavioral problems to be due to road traffic noise at night. This suggests the importance of assessing the effect of nighttime noise levels, in addition to daytime residential and school noise levels. Future research could be improved by assessing both classroom and environmental noise, using standard methods and metrics, and by using commonly used validated outcome assessments.

The observed effects of traffic noise are sometimes small, but because traffic noise is ubiquitous and inescapable in many residential areas, these small effects are relevant to public health. However, measuring small effects requires large sample sizes that are often impossible to acquire with individual studies. Using standard methods for assessing the effects of environmental noise would make it possible to pool the data of individual studies in order to achieve the necessary statistical power. In the area of environmental noise research, individual study data has been pooled before examining the relationship between nighttime noise and sleep disturbances [55]. A pooled analysis could provide a more precise understanding of the relationship between environmental noise and children’s mental health.

Although we excluded studies that considered non-clinical health outcomes such as annoyance and well-being, these are also important health indicators to consider. Noise-related annoyance may be an intermediate factor between traffic noise-exposures and the development of behavioral or emotional disorders. A systematic review by van Kamp and Davies [56] considered noise-annoyance among children (among other outcomes), and found only two studies considering traffic-related noise annoyance among children. A more recent narrative review by Stansfeld and Clark [48] describes only one further study on traffic-related noise annoyance among children. While traffic-noise related annoyance has been widely researched, it appears to be less well researched in children.

All of the included studies adjusted for household indicators of social and material deprivation (i.e., social-economic status), but few of the included studies considered the impact of other environmental factors that may also be present in residential areas exposed to traffic noise, such as air pollution and a lack of green spaces. Only two studies adjusted for air pollution [37,41], and two further studies considered air pollution in additional analyses [40,44]. Only Forns et al. [41] adjusted for urban vulnerability, and none of the studies considered the amount of green spaces in the residential areas. Vulnerable populations often live in neighborhoods with poorer environmental and social conditions, and these exposures may also be contributing to a mental health risk among already vulnerable children [57]. Promoting environmental justice by reducing sources of noise and air pollution, increasing access to green spaces, and improving the infrastructure in socially vulnerable neighborhoods may also help improve the mental health of children living in these neighborhoods.

## 5. Conclusions

Children have very little control or choice regarding their place of residence, and may be less able to identify, comprehend, or effectively deal with potential sources of stress such as transportation noise. They also have an increased need for sleep, which is important for their physical growth and cognitive development. Annoyance and sleep disturbances due to transportation noise may be increasing risks of emotional and behavioral problems among children. However, a majority of the existing research has focused on traffic noise exposure at school. While transportation noise levels at school may be correlated with traffic noise levels at homes, research from birth cohorts considering (long-term) noise exposures at home were more likely to find significant relationships between noise and hyperactivity and inattention problems. Since most of the existing evidence has concentrated on the noise exposures at school, further research on residential childhood (nighttime) traffic noise exposure is needed to determine if the risk of conduct disorders is indeed increased by transportation noise. Additionally, using standardized study methods to conduct future research should make it possible to pool individual study data, which may be necessary to obtain precise effect estimates for environmental noise and children’s mental health.

## Figures and Tables

**Table 1:** Study inclusion & exclusion criteria.

Category	Inclusion	Exclusion
Population	Children sampled from the general population	Animal studies; occupational populations, non-representative (i.e., convenience) samples
Exposure	Road traffic, railway or aircraft noise assessed objectively (i.e., measurements or noise modelling at place of residence or at school)	Military aircraft noise; industrial noise; studies considering only neighborhood noise
Outcomes	Behavioral and emotional disorders in children (ICD-10 F90–F98) (i.e., diagnosed disorders, e.g., self-reported, routine data; prescribed medications specific to a psychological disorder; validated screening instrument)	Annoyance; sleep disturbance; conditions not directly related to a clinical diagnosis; screening instrument was not validated

**Table 2:** Studies of childhood behavioral disorders.

Study	Region(s) Study Design Quality Score (++,+,-)	Population	Outcome Assessment	Noise Source/Location	Noise Parameters	Noise Categories	Effect Estimates; Meta-Analysis (Yes, No With Reasons)
Belojevic et al. 2012 [42]	Serbia cross-sectional (-)	*N* = 311 (M = 146, F = 165) 7–11 Years	Adapted ADD Questionnaire	Road home and at school	calculated L_24h_	Continuous analysis	Beta (adjusted) no: continuous endpoints
Clark et al. 2013 [32]	UK cohort (-)	*N* = 461 (M = 202, F = 259) 15–17 Years	SDQ	aircraft road at school	L_eq, 07–23h_	Continuous analysis	Beta (adjusted) no: continuous endpoints
Crombie et al. 2011 [33] with Stansfeld et al. 2005 [43], Clark et al. 2012 [44]	UK, Spain, NL cross-sectional (-)	*N* = 1900 (M = 897, F = 1003) Mean Age: 10.6 Years	SDQ	aircraft road at school	L_eq, 07–23h_	Continuous analysis	Beta (adjusted) no: continuous endpoints
Forns et al. 2016 [41]	Spain cross-sectional (-)	*N* = 2897 (M = 1446, F = 1430) 7–11 Years	SDQADHD-DSM-IV	traffic in one classroom per school	-	Continuous analysis	Adjusted mean ratio no: continuous endpoints
Haines et al. 2001 [34,35,36]	UK cohort/ cross-sectional (+)/(-)	*N* = 275/*N* = 451 (M = 143, F = 132) 10 Years	SDQCDICMAS	aircraft at school	L_eq, 07–23h_	High noise: L_eq,23–07h_ > 63 dBL _eq,23–07h_ ≥ 66 dB	Averages (matched) no: only group averages reported
Hjortebjerg et al. 2016 [37]	Denmark cohort (+)	*N* = 46,940 7 years	SDQ	Road ^a^ railway ^b^ at home	L_eq,23–07h_ L_DEN_	Continuous (per 10 dB)	Odds Ratio (adjusted) yes
Lim et al. 2018 [38]	South Korea cross-sectional (-)	*N* = 918 (M = 427, F = 491) Mean Age: 11.5 years	CBCL	roadat school	L_DN_	Continuous (per 5 dB)	Odds Ratio(adjusted) yes
Stansfeld et al. 2009 [39]	UK, Spain, NL cross-sectional (-)	*N* = 2014 9–10 years	SDQ	airwayroadat school	L_eq, 07–23h_	Continuous analysis	Beta (adjusted) no: continuous endpoints
Tiesler et al. 2013 [20]	Germany Cross-sectional (-)	*N* = 872 (M = 410, F = 462) 10 years	SDQ	roadat home	L_eq,22–06h_^c^ L_DEN_	Continuous (per IQR ^d^)	Odds Ratio (adjusted) yes
Weyde et al. 2018 [40]	Norwaycohort (+)	*N* = 1934 (prenatal) *N* = 1384 (postnatal) M = 47.5–52.5%	RSDBD	Road ^e^ at home	L_DEN_	Continuous analysis	Beta (adjusted) no: continuous endpoints

ADD Attention Deficit Disorder; SDQ Strengths and Difficulties Questionnaire; CBCL Child Behavior Checklist; CDI Child Depression Inventory; CMAS Child Manifest Anxiety Scale; RSDBD Rating Scale for Disruptive Behavior Disorders. ^a^ road noise levels starting from 40 dB, noise under dB set to 40 dB. ^b^ railway noise starting from 0 dB, noise under 20 dB set to 0 dB. ^c^ lowest L_eq,22-06h_ value 26.9 dB; lowest L_DEN_ at most exposed house facade 35.5 dB. ^d^ IQR: interquartile range (ca. 8.2 dB to 9 dB). ^e^ Railway noise was also estimated, but the categories of railway noise were considered as an adjustment variable.

**Table 3:** Summary of the results for childhood behavioral disorders.

		Depression	Anxiety	Hyperactivity/Inattention	Conduct Problems	Emotional Symptoms	Peer Problems	Psychosocial Behavior	SDQ/CBCL Total ^a^
*Noise at school*									
Belojevic 2012 (road)	continuous			↑ ^b^					O
Clark 2013 (aircraft)	β per 1 dB			O	O	O			O
Crombie et al. 2011 with Stansfeld et al. 2005 [43], Clark et al. 2012 [44] (aircraft/road)	β per 1 dB			Air (↑)/Road O	Air O/Road ↓	Air O/Road O			Air O/Road O
Forns 2016 (‘traffic’)	continuous			↑ ^c^					
Haines 2001 (a,b,c) (aircraft)	Mean diff. High v. low	(a) O/(b) O	(a) O/(b) O	(a) O/(c) ↑	(a) O/(c) O	(a) O/(c) O	(a) O/(c) O	(a) O	(a) O/(c) ↑
Lim 2018 (road)	per 1 dB				O ^d^	O ^d^			↑ ^d^
Stansfeld 2009 (aircraft/road)	β per 1 dB			Air ↑/Road O	Air O/Road ↑	Air O/Road O	Air O/Road O		Air O/Road O
*Noise at home*									
Hjortebjerg 2016 (road/railway) ^e^	OR per 10 dB			Road ↑/Rail O	Road O/Rail O	Road O/Rail O	Road O/Rail ↑		
Tiesler 2013 (road)	OR per 10 dB			↑	O	O	O		O
Weyde 2017 (road) ^e^	fractional logit (continuous)			↑					

O non-significant association; ↑ statistically significant increase/increased risk; ↓ statistically significant decrease/decreased risk; (↑) borderline significant increase (*p* = 0.05); Air = aircraft noise; Road = road traffic noise; Rail = railway noise. ^a^ SDQ sum without prosocial psychosocial behavior scale or sum of CBCL scales. ^b^ Adapted version of the Attention Deficit Disorder Questionnaire (5 item scale). ^c^ ADHD-DSM-IV list. ^d^ CBCL checklist: conduct problems = externalizing problems; emotional symptoms = internalizing problems. ^e^ Results for noise exposure up to or at follow-up (at age 7 or 8). These studies also considered noise exposures during pregnancy or at birth, and found no clear associations between these pre-natal/natal noise exposures and later behavioral problems.

## Supplementary Materials

The following are available online at https://www.mdpi.com/1660-4601/16/18/3336/s1, Table S1: Studies excluded with reasons, Table S2: Detailed extraction table of 10 studies (14 publications).
