# Supplementary material for: Behavioral and Emotional Disorders and Transportation Noise among Children and Adolescents: A Systematic Review and Meta-Analysis
Source: Int J Environ Res Public Health. 2019 Sep 10;16(18):3336. doi: 10.3390/ijerph16183336 (PMC6765874; doi:10.3390/ijerph16183336)
Supplement: Supplementary file 1 [file ijerph-16-03336-s001.pdf]

Table S1. Studies excluded with reasons

Outcome not (clinical) mental disorder (n = 99)

1. Al-Mutairi, N. Z., Al-Attar, M. A., & Al-Rukaibi, F. S. (2011). Traffic-generated noise pollution: exposure of road users and populations in Metropolitan Kuwait. *Environmental Monitoring & Assessment*, *183*(1-4), 65-75. doi: 10.1007/s10661-011-1906-0
2. Arbeitsgemeinschaft für sozio-psychologische Fluglärmuntersuchungen (1973): Untersuchungen über den Fluglärm und seine Wirkungen im Gebiet von drei Schweizer Zivilflughäfen 1971/72. Eidgenössisches Luftamt, Bundeshaus, Bern, Schweiz.
3. Babisch, W., Schulz, C., Seiwert, M., & Conrad, A. (2012). Noise annoyance as reported by 8-to 14-year-old children. *Environment &Behavior*, *44*(1), 68-86. doi: 10.1177/0013916510387400
4. Barceló, M. A., Varga, D., Tobias, A., Diaz, J., Linares, C., & Saez, M. (2016). Long term effects of traffic noise on mortality in the city of Barcelona, 2004–2007. *Environmental Research*, *147*, 193-206. doi: 10.1016/j.envres.2016.02.010
5. Basner, M., & Samel, A. (2004). Nocturnal aircraft noise effects. *Noise and Health*, *6*(22), 83.
6. Bättig, K. & Buzzi, R. (1979). Psychophysiological effects of aircraft noise. *Activitas Nervosa Superior*, 21(4), 257-258.
7. Bättig, K., Zeier, H., Müller, R., & Buzzi, R. (1980). A field study on vegetative effects of aircraft noise. *Archives of Environmental Health: An International Journal*, *35*(4), 228-235.
8. Bättig, K., & Buzzi, R. (1981). Psychophysiologische Effekte von Lärm und Beschäftigung in der Heimsituation. *Zeitschrift für Experimentelle und Angewandte Psychologie*, *28*, 1-14.
9. Bättig, K., & Buzzi, R. (1981). Psychophysiological effects of noise and activity in the home situation. *Zeitschrift für Experimentelle und Angewandte Psychologie*, *28*, 1-14.
10. Bättig, K., & Buzzi, R. (1982). Physiological responses to noise and to the type of activity under field conditions. *Activitas Nervosa Superior*, (Pt 1), 236-240.
11. Baumbach, W., Mörstedt, R., Schulze, B., Wölke, G., Ullmann, R., & Grossmann, G. (1990). New aspects of the traffic noise problem in the inner city area. *Zeitschrift für die gesamte Hygiene und ihre Grenzgebiete*, *36*(4), 204-206.
12. Beutel, M. E., Jünger, C., Klein, E. M., Wild, P., Lackner, K., Blettner, M., ... & Münzel, T. (2016). Noise annoyance is associated with depression and anxiety in the general population-the contribution of aircraft noise. *Plos one*, *11*(5), e0155357. doi: 10.1371/journal.pone.0155357
13. Birnie, S. E., Hall, F. L., & Taylor, S. M. (1980). Community response to noise from a general aviation airport. *Noise Control Eng.;(United States)*, *15*.
14. Björk, J., Ardö, J., Stroh, E., Lövkvist, H., Östergren, P. O., & Albin, M. (2006). Road traffic noise in southern Sweden and its relation to annoyance, disturbance of daily activities and health. *Scandinavian Journal of Work, Environment & Health*, 32(5), 392-401. doi:10.5271/sjweh.1035
15. Björk, J., Ardö, J., Stroh, E., Lövkvist, H., Östergren, P. O., & Albin, M. (2007). Erratum: Road traffic noise in southern Sweden and its relation to annoyance, disturbance of daily activities and health. *Scandinavian Journal of Work, Environment & Health*, 33(1), 392-401.
16. Black, D. A., Black, J. A., Issarayangyun, T., & Samuels, S. E. (2007). Aircraft noise

exposure and resident's stress and hypertension: A public health perspective for airport environmental management. *Journal of Air Transport Management*, *13*(5), 264-276. doi: 10.1016/j.jairtraman.2007.04.003
17. Bodenheim, A. & Unger, M (1991). Psychosoziale Auswirkungen des Wohnens an hochfrequentierten Straßen : Eine Erkundungsstudie. *Unser Doppelleben. Neue Studien zur Umweltbetroffenheit,* 103-139.
18. Botteldooren, D., Dekoninck, L., & Gillis, D. (2011). The influence of traffic noise on appreciation of the living quality of a neighborhood. *International Journal of Environmental Research and Public Health*, *8*(3), 777-798. doi: 10.3390/ijerph8030777
19. Brink, M. (2011). Parameters of well-being and subjective health and their relationship with residential traffic noise exposure—A representative evaluation in Switzerland. *Environment International*, *37*(4), 723-733. doi: 10.1016/j.envint.2011.02.011.
20. Brown, A. L. (1984). Critique of subjective responses of Chinese to aircraft noise. *Applied Acoustics*, *17*(3), 223-232.
21. Bullinger, M., & Bahner, U. (1997). Erlebte Umwelt und subjektive Gesundheit. Eine Untersuchung an Müttern und Kindern aus unterschiedlichen lärmbelasteten Gebieten. *Zeitschrift fur Gesundheitwissenschaften*, *3*, 89-108.
22. Bullinger, M., Hygge, S., Evans, G. W., Meis, M., & Mackensen, S. V. (1999). The psychological cost of aircraft noise for children. *Zentralblatt für Hygiene und Umweltmedizin*, *202*(2-4), 127-138. doi: 10.1080/00207594.2013.804190
23. Bullinger, M. (2000). Wahrgenommene Umweltbelastung und gesundheitsbezogene Lebensqualität von Müttern und Kindern : Eine umweltepidemiologische Untersuchung an Familien aus unterschiedlich fluglärmbelasteten Gebieten in Bayern. *Lebensqualitätsforschung aus medizinpsychologischer und -soziologischer Perspektive*, 354-367.
24. Buzzi, R., & Bättig, K. (1984). Extraaurale beziehungsweise vegetative Effekte von Umweltlärm. *Beiträge zur Bedeutungslehre des Schalls. Peter Lang Verlag, Bern*, 243-251.
25. Carter, N. L. (1996). Transportation noise, sleep, and possible after-effects. *Environment International*, *22*(1), 105-116. doi: 10.1016/0160-4120(95)00108-5
26. Clark, C. & Sörqvist, P. (2012). The influence of noise on performance and behavior. *Noise Health*, 14, 292–296.
27. Conzelmann-Auer, C., Braun-Fahrländer, C., Ackermann-Liebrich, U., & Wanner, H. U. (1993). Perception of traffic noise emission in Basel city canton in comparison to actually measured noise levels. *Sozial-und Präventivmedizin*, *38*(4), 231-238. doi: 10.1007/BF01624541
28. De Jong, R. G. (1979). A Dutch study on railroad traffic noise. *Journal of Sound and Vibration*, *66*(3), 497-502. doi: 10.1016/0022-460X(79)90871-X
29. De Jong, R. G. (1981). Dutch study of noise disturbance due to airplanes. *Geluid Omgeving*, *4*(4), 228-232.
30. De Kluizenaar, Y. , Salomons, E. M., Janssen, S. A., van Lenthe, F. J., Vos, H., Zhou, H., ... & Mackenbach, J. P. (2011). Urban road traffic noise and annoyance: The effect of a quiet façade. *The Journal of the Acoustical Society of America*, *130*(4), 1936-1942. doi: 10.1121/1.3621180.
31. de Paiva Vianna, K. M., Cardoso, M. R. A., & Rodrigues, R. M. C. (2015). Noise pollution and annoyance: An urban soundscapes study. *Noise & Health*, *17*(76), 125. doi: 10.4103/ 1463-1741.155833

32. Delauzun, F. R., & Griffiths, I. D. (1978). The problem of individual differences in sensitivity to traffic noise and the establishment of standards. *International Review of Applied Psychology*. doi: 10.1111/j.1464-0597.1978.tb00354.x
33. Dratva, J., Zemp, E., Dietrich, D. F., Bridevaux, P. O., Rochat, T., Schindler, C., & Gerbase, M. W. (2010). Impact of road traffic noise annoyance on health-related quality of life: Results from a population-based study. *Quality of Life Research*, *19*(1), 37-46. doi: 10.1007/s11136-009-9571-2
34. Evans, G. W., Hygge, S., & Bullinger, M. (1995). Chronic noise and psychological stress. *Psychological Science*, *6*(6), 333-338. doi: 10.1111/j.1467-9280.1995.tb00522.x
35. Evans, G. W., Bullinger, M., & Hygge, S. (1998). Chronic noise exposure and physiological response: A prospective study of children living under environmental stress. *Psychological Science*, *9*(1), 75-77. doi: 10.1111/1467-9280.00014
36. Fiedler, F. E., & Fiedler, J. (1975). Port noise complaints: Verbal and behavioral reactions to airport-related noise. *Journal of Applied Psychology*, *60*(4), 498. Doi: 10.1037/h0076924
37. Findeis, H., & Peters, E. (2004). Disturbing effects of low frequency sound immissions and vibrations in residential buildings. *Noise and Health*, *6*(23), 29.
38. Fooladi, M. M. (2012). Involuntary and persistent environmental noise influences health and hearing in Beirut, Lebanon. *Journal of Environmental and Public Health*, 2012: 235618. doi: 10.1155/2012/235618
39. Franssen, E. A. M., Van Wiechen, C. M. A. G., Nagelkerke, N. J. D., & Lebret, E. (2004). Aircraft noise around a large international airport and its impact on general health and medication use. *Occupational and Environmental Medicine*, *61*(5), 405-413. doi: 10.1136/oem.2002.005488
40. Furlan, P. M., Bergamini, L. & Bergamasco, B. (1976). Street noise. A psychologic and neurophysiologic study. *Rassegna Di Studi Psichiatrici*, *65*: 519-530.
41. Gómez-Jacinto, L., & Moral-Toranzo, F. (1999). Urban traffic noise and self-reported health. *Psychological Reports*, *84*(3_suppl), 1105-1108. doi:10.2466/pr0.1999.84.3c.1105
42. Goswami, S., Nayak, S. K., Pradhan, A. C., & Dey, S. K. (2011). A study on traffic noise of two campuses of University, Balasore, India. *Journal of Environmental Biology, 32*(1), 105-109.
43. Gozalo, G. R., & Barrigón Morillas, J. M. (2016). Analysis of sampling methodologies for noise pollution assessment and the impact on the population. *International Journal of Environmental Research and Public Health*, *13*(5), 490. doi: 10.3390/ijerph13050490
44. Graeven, D. B. (1974). The effects of airplane noise on health: An examination of three hypotheses. *Journal of Health and Social Behavior*, *15*(4), 336-343. doi: 10.2307/2137094
45. Greiser, E. (2014). Health risk railroad noise-prognosis of potential health risks subsequent to night-time exposure to railroad noise in the German part of the Transversal Rotterdam Genova. *Gesundheitswesen (Bundesverband der Ärzte des Öffentlichen Gesundheitsdienstes (Germany))*, *76*(12), 862-864. doi: 10.1055/s-0034-1395639
46. Gundersen, H., Magerøy, N., Moen, B. E., & Bråtveit, M. (2013). Traffic density in area of residence is associated with health-related quality of life in women, the community-based Hordaland Health Study. *Archives of Environmental & Occupational Health*, *68*(3), 153-160. doi: 10.1080/19338244.2012.676103
47. Haines, M. M., Brentnall, S. L., Stansfeld, S. A., & Klineberg, E. (2003). Qualitative responses of children to environmental noise. *Noise and Health*, *5*(19), 19.

48. Hammad, R. N. S., & Abdelazeez, M. K. (1987). Measurements and analysis of the traffic noise in Amman, Jordan and its effects. *Applied Acoustics*, *21*(4), 309-320. doi: 10.1016/0003-682X(87)90052-1
49. Hammersen, F., Niemann, H., & Hoebel, J. (2016). Environmental noise annoyance and mental health in adults: findings from the cross-sectional German Health Update (GEDA) Study 2012. *International Journal of Environmental Research and Public Health*, *13*(10), 954. doi:10.3390/ijerph13100954
50. Han, Z. X., Lei, Z. H., Zhang, C. L., Xiong, W., Gan, Z. L., Hu, P., & Zhang, Q. B. (2015). Noise monitoring and adverse health effects in residents in different functional areas of Luzhou, China. *Asia Pacific Journal of Public Health*, *27*(2_suppl), 93S-99S. doi: 10.1177/1010539514560056
51. Hawkins, M. M., & Large, J. B. (1983). Subjective response to noise in rural villages, particularly from road traffic. *Journal of Sound and Vibration*, *88*(3), 321-331. doi: 10.1016/0022-460X(83)90691-0
52. Héritier, H., Vienneau, D., Frei, P., Eze, I. C., Brink, M., Probst-Hensch, N., & Röösli, M. (2014). The association between road traffic noise exposure, annoyance and health-related quality of life (HRQOL). *International Journal of Environmental Research and Public Health*, *11*(12), 12652-12667. doi: 10.3390/ijerph111212652
53. Honold, J. & van der Meer, E. (2017). Residential exposure to multiple environmental burdens and health: A mixed-methods study**.** *Umweltpsychologie* 21(1), 119-138.
54. Jarup, L., Dudley, M. L., Babisch, W., Houthuijs, D., Swart, W., Pershagen, G., ... & Vigna-Taglianti, F. (2005). Hypertension and exposure to noise near airports (HYENA): study design and noise exposure assessment. *Environmental Health Perspectives*, *113*(11), 1473. doi:10.1289/ehp.8037
55. Jenkins, M. A., & Pahl, J. (1975). Measurement of freeway noise and community response. *The Journal of the Acoustical Society of America*, *58*(6), 1222-1231. doi: 10.1121/1.380804
56. Karsdorf, G., & Klappach, H. (1968). Einflüsse des Verkehrslärms auf Gesundheit und Leistung bei Oberschülern einer Großstadt. *Zeitschrift für die Gesamte Hygiene und ihre Grenzgebiete*, *14*, 52-54.
57. Kastka, J. (1984). Untersuchungen zur Belästigung durch Verkehrslärm: umweltpsychiologische Analysen zu einem umwelthygienischen Konzept. Dissertation Universität Düssldorf.
58. Klatte, M., Spilski, J., Mayerl, J., Möhler, U., Lachmann, T., & Bergström, K. (2017). Effects of aircraft noise on reading and quality of life in primary school children in Germany: Results from the NORAH study. *Environment and Behavior*, *49*(4), 390-424. doi: 10.1177/0013916516642580
59. Knipschild, P. (1977). V. Medical effects of aircraft noise: community cardiovascular survey. *International Archives of Occupational and Environmental Health*, *40*(3), 185-190. doi : 10.1007/BF01842081
60. Ko, J. H., Chang, S. I., Kim, M., Holt, J. B., & Seong, J. C. (2011). Transportation noise and exposed population of an urban area in the Republic of Korea. *Environment International*, *37*(2), 328-334. doi: 10.1016/j.envint.2010.10.001
61. Koszarny, Z. (2001). The estimate of well-being and self-assessed health status in urban population in various acoustic areas. *Roczniki Panstwowego Zakladu Higieny*, *52*(2), 165-178.
62. Koszarny, Z., Szata, W., & Goryński, P. (1979). Exposure to railway noise and its

effect on the population. *Roczniki Panstwowego Zakladu Higieny*, *30*(4), 387-395.
63. Kryter, K. D. (2009). Acoustical model and theory for predicting effects of environmental noise on people. *The Journal of the Acoustical Society of America*, *125*(6), 3707-3721. doi: 10.1121/1.3125320
64. Krert, E. (1986). Entstehung und Bewältigung von Lärmbelastungen - insbesondere Fluglärmbelastungen - bei Kindern. Dissertation Universität Graz.
65. La Torre, G., Moscato, U., La Torre, F., Ballini, P., Marchi, S., & Ricciardi, W. (2007). Environmental noise exposure and population health: a cross-sectional study in the Province of Rome. *Journal of Public Health*, *15*(5), 339-344. doi: 10.1007/s10389-007-0144-y
66. Lercher, P., & Kofler, W. W. (1996). Behavioral and health responses associated with road traffic noise exposure along alpine through-traffic routes. *Science of the Total Environment, 189-190*, 85-89 doi: 10.1016/0048-9697(96)05194-7
67. Lercher, P., Evans, G. W., Meis, M., & Kofler, W. W. (2002). Ambient neighbourhood noise and children's mental health. *Occupational and environmental medicine*, *59*(6), 380-386. doi: 10.1136/oem.59.6.380
68. Lefèvre, M., Carlier, M. C., Champelovier, P., Lambert, J., Laumon, B., & Evrard, A. S. (2017). Effects of aircraft noise exposure on saliva cortisol near airports in France. *Occupational Environmental Medicine*, oemed-2016. doi: 10.1136/oemed-2016-104208
69. Maschke, C. (2003). Epidemiological research on stress caused by traffic noise and its effects on high blood pressure and psychic disturbances. In *Proceedings of the 8th International Congress on Noise as a Public Health Problem (Jong RD, Houtgast T, Franssen EAM, Hofman W, eds). Shiedam, the Netherlands: Foundation ICBEN* (pp. 96-101).
70. Matheson, M. P., Stansfeld, S. A., & Haines, M. M. (2003). The effects of chronic aircraft noise exposure on children's cognition and health: 3 field studies. *Noise and Health*, *5*(19), 31.
71. Meister, E. A., & Donatelle, R. J. (2000). The impact of commercial-aircraft noise on human health: a neighborhood study in metropolitan Minnesota. *Journal of Environmental Health*, *63*(4), 9.
72. Mosskov, J. I., & Ettema, J. H. (1977). IV. Extra-auditory effects in long-term exposure to aircraft and traffic noise. *International Archives of Occupational and Environmental Health*, *40*(3), 177-184. doi: 10.1007/BF01842080
73. Müller, R. (1980). Does aircraft noise affect health? *Sozial-und Präventivmedizin*, *25*(3), 103-109. doi: 1007/BF02074972
74. Noack, R. H. (1995). *Lärm und Ruhe: Untersuchungen zur Belästigung durch Verkehrsgeräusche und Pegelschwankungen*. dis's' kurs.
75. Pedersen, E. (2015). City dweller responses to multiple stressors intruding into their homes: Noise, light, odour, and vibration. *International Journal of Environmental Research and Public Health*, *12*(3), 3246-3263. doi: 10.3390/ijerph120303246
76. Pulles, M. P. J., Biesiot, W., & Stewart, R. (1990). Adverse effects of environmental noise on health: an interdisciplinary approach. *Environment International*, *16*(4-6), 437-445. doi: 10.1016/0160-4120(90)90012-U
77. Relster, E. (1977). Influence of traffic noise *on* mental health. *Ugeskr Laeger*, 139 (9):549-552.
78. Rilfatti, M. (1982). Human reactions to community noise. I. Noise of urban street traffic in the city of Verona. *Nuovi Annali d'Igiene e Microbiologia*, *33*(4-6), 799-815.
79. Röösli, M., Vienneau, D., & Perez, L. (2015). Transportation noise:'neglected

exposure'on a global scale. *Tropical Medicine & International Health*, *20*, 4.
80. Schreckenberg, D., & Meis, M. (2006). Effects of aircraft noise on noise annoyance and quality of life around Frankfurt Airport. Final abridged report
81. Schreckenberg, D., Griefahn, B., & Meis, M. (2010). The associations between noise sensitivity, reported physical and mental health, perceived environmental quality, and noise annoyance. *Noise and Health*, *12*(46), 7. doi: 10.4103/1463-1741.59995.
82. Schreckenberg, D., Meis, M., Kahl, C., Peschel, C., & Eikmann, T. (2010). Aircraft noise and quality of life around Frankfurt Airport. *International Journal of Environmental Research and Public Health*, *7*(9), 3382-3405. doi: 10.3390/ijerph7093382
83. Seabi, J. (2013). An epidemiological prospective study of children's health and annoyance reactions to aircraft noise exposure in South Africa. *International Journal of Environmental Research and Public Health*, *10*(7), 2760-2777. doi: 10.3390/ijerph10072760
84. Seidman, M. D., & Standring, R. T. (2010). Noise and quality of life. *International Journal of Environmental Research and Public Health*, *7*(10), 3730-3738. doi: 10.3390/ijerph7103730
85. Shepherd, D., Dirks, K., Welch, D., McBride, D., & Landon, J. (2016). The covariance between air pollution annoyance and noise annoyance, and its relationship with health-related quality of life. *International Journal of Environmental Research and Public Health*, *13*(8), 792. doi: 10.3390/ijerph13080792

86. Stansfeld, S., Hygge, S., Clark, C., & Alfred, T. (2010). Night time aircraft noise exposure and children's cognitive performance. *Noise and Health*, *12*(49), 255. doi: 10.4103/1463-1741.70504.
87. Stansfeld, S. A., & Shipley, M. (2015). Noise sensitivity and future risk of illness and mortality. *Science of the Total Environment*, *520*, 114-119. doi: 10.1016/j.scitotenv.2015.03.053.
88. Tzivian, L., Foraster, M., Hennig, F.,Fuks, K., Sugiri, D.,Erbel, R., Jockel, H. K., Moebus, S.& Hoffmann, B. (2016). Long-term traffic noise exposure corrected for indoors and its association with outdoor air pollution and annoyance in participants of the Heinz Nixdorf recall study. *European Journal of Epidemiology,* 31, S214-S215. doi: 10.1289/ehp.1509824
89. Urban, J., & Máca, V. (2013). Linking traffic noise, noise annoyance and life satisfaction: A case study. *International Journal of Environmental Research and Public Health*, *10*(5), 1895-1915. doi: 10.3390/ijerph10051895
90. van den Berg, F., Verhagen, C., & Uitenbroek, D. (2015). The relation between self-reported worry and annoyance from air and road traffic. *International Journal of Environmental Research and Public Health*, *12*(3), 2486-2500. doi: 10.3390/ijerph120302486
91. van Kempen, E., van Kamp, I., Nilsson, M., Lammers, J., Emmen, H., Clark, C., & Stansfeld, S. (2010). The role of annoyance in the relation between transportation noise and children's health and cognition. *The Journal of the Acoustical Society of America*, *128*(5), 2817-2828. doi: 10.1121/1.3483737
92. Van Wiechen, C. M., Franssen, E. A., de Jong, R. G., & Lebret, E. (2002). Aircraft noise exposure from Schiphol airport: a relation with complainants. *Noise and Health*, *5*(17), 23.
93. Ward, L. M., & Suedfeld, P. (1973). Human responses to highway noise. *Environmental Research*, *6*(3), 306-326. doi: 10.1016/0013-9351(73)90043-1

94. Waters, D. M. (1979). Overall railway noise impact in the UK. *Journal of Sound and Vibration*, *66*(3), 477-481 doi: 10.1016/0022-460X(79)90868-X
95. Welch, D., Shepherd, D., Dirks, K. N., McBride, D., & Marsh, S. (2013). Road traffic noise and health-related quality of life: A cross-sectional study. *Noise and Health*, *15*(65), 224. doi: 10.4103/1463-1741.113513.
96. Wothge, J., Belke, C., Möhler, U., Guski, R., & Schreckenberg, D. (2017). The Combined Effects of Aircraft and Road Traffic Noise and Aircraft and Railway Noise on Noise Annoyance—An Analysis in the Context of the Joint Research Initiative NORAH. *International Journal of Environmental Research and Public Health*, *14*(8), 871. doi: 10.3390/ijerph14080871.
97. Yokoo, Y., & Mitani, Y. (1982). The effects of chronic exposure to train noise on the mental efficiency of elementary school children (report 2). *Nippon Eiseigaku Zasshi (Japanese Journal of Hygiene)*, *37*(5), 753-761. doi: 10.1265/jjh.37.753
98. Yoshida, T., & Nakamura, S. (1988). Subjective ratings of health status and railway noise. *Journal of Sound and Vibration*, *127*(3), 593-598. doi: 10.1016/0022-460X(88)90388-4
99. Zijlema, W. L., Morley, D. W., Stolk, R. P., & Rosmalen, J. G. M. (2015). Noise and somatic symptoms: A role for personality traits?. *International Journal of Hygiene and Environmental Health*, *218*(6), 543-549. doi: 0.1016/j.ijheh.2015.05.001

Reviews/ Letters/ Editorials (n = 51)

1. Adams, L. (2017). Dementia: Could heavy traffic drive dementia risk?. *Nature Reviews Neurology*, *13*(3), 128. doi: 10.1038/nrneurol.2017.6
2. Anonymous (1979). Minds under the flightpath. *Lancet*, 2(8156-8157), 1343.
3. Anonymous (1992). Health effects of the noise of low-flying aircrafts. [German]. *Zeitschrift für Lärmbekämpfung*, 39(2), 52-54.
4. Anonymous (2002). Noise hurts boys and girls. *Environmental Health Perspectives*, 110(10), A569.
5. Babisch, W. (1985). Danger to health caused by environmental noise?. *Schriftenreihe des Vereins für Wasser-, Boden-und Lufthygiene*, *65*, 425.
6. Babisch, W. (2000). Health effects of traffic noise. [German]. *Schriftenreihe des Vereins für Wasser-, Boden-und Lufthygiene*, 106, 178-192.
7. Babisch, W. (2004). Health aspects of extra-aural noise research. *Noise and Health*, *6*(22), 69.
8. Björklid, P. (1994). Children-traffic-environment. *Architecture & Comportement*, *10*(4), 361-369.
9. Bröer, C. (2007). Aircraft noise and risk politics. *Health, Risk & Society*, *9*(1), 37-52. https://doi.org/10.1080/13698570601181631
10. Camard, J. P., Lefranc, A., Gremy, I., & Ferry, R. (2004). Noise-induced health effects: Recent epidemiological studies. *Environnement, Risques & Santé*, *3*(4), 235-242.
11. Cho, H. W. & Chu, C. (2014). Sound in the air. Osong Public Health and Research Perspectives, 5(2): 75-76. doi: 10.1016/j.phrp.2014.04.001
12. Chowns, R. H., Abey-Wickrama, I., A'Brook, M. F., Gattoni, F. E. G., & Herridge, C. F. (1970). Mental-hospital admissions and aircraft noise. *The Lancet*, *295*(7644), 467-468. doi: 10.1016/S0140-6736(70)90858-5
13. Dalle, B. (1966). Troubles du comportement et vie dans les nouveaux ensembles urbains. *L'Évolution Psychiatrique*.
14. Dora, C., & Phillips, M. (Eds.). (2000). *Transport, environment and health* (No. 89). WHO

Regional Office Europe.
15. Esser, A. H. (1974). Environment and mental health. *Science, Medicine and Man*, *1*(3), 181-193.
16. Evans, G. W. (2003). The built environment and mental health. *Journal of Urban Health, 80*(4), 536-555. doi:10.1093/jurban/jtg063
17. Gottlob, D. & Meurers, H. (1984). Effects of road traffic noise. Umweltpolitik 7 (1), 41-59.
18. Haines, M., & Stansfeld, S. (2003). Ambient neighbourhood noise and children's mental health. *Occupational and Environmental Medicine*, *60*(2), 146-146. doi: 10.1136/oem.60.2.146
19. Health Council of the Netherlands: Committee on the Health Impact of Large Airports. Public Health
20. Herridge, C. F. (1974). Aircraft noise and mental health. *Journal of Psychosomatic Research, 18*(4), 239-243. doi: 10.1016/0022-3999(74)90004-X
21. Impact of Large Airports. The Hague: Health Council of the Netherlands, 1999 (1999/14E).
22. Ising, H., Babisch, W., & Kruppa, B. (1998). Ergebnisse epidemiologischer Forschung im Bereich Lärm. In Gesundheitsrisiken durch Lärm-Tagungsband zum Symposium Veranstaltungen im Rahmen der Initiative „Schritte zu einer nachhaltigen, umweltgerechten Entwicklung". Wissenschaftszentrum Bonn, 10. Februar 1998.
23. Jansen, G. (1986). Zur „erheblichen Belästigung "und „Gefährdung "durch Lärm. *Zeitschrift für Lärmbekämpfung, 33*, 2-7.
24. Krichagin, V. J. (1978). Health effects of noise exposure. *Journal of Sound and Vibration, 59*(1), 65-71. doi: 10.1016/0022-460X(78)90479-0
25. Kryter, K. D. (1966). Psychological reactions to aircraft noise. *Science,* 151(716), 1346-1355. doi: 10.1126/science.151.3716.1346
26. Kryter, K. D. (1968). An example of" engineering psychology": The aircraft noise problem. *American Psychologist*, *23*(4), 240. doi: 10.1037/h0026248
27. Kumar, D. (2010). Noise pollution in India. *Journal of the Indian Medical Association*, 108(3), 139.
28. McLean, E. K., & Tarnopolsky, A. (1977). Noise, discomfort and mental health: a review of the socio-medical implications of disturbance by noise. *Psychological Medicine*, *7*(1), 19-62. doi: 10.1017/S0033291700023138
29. Meyer, A. (2003). Wenn kaum noch jemand weiter weiß. Eine repräsentative sozio-psychologische Fluglärmuntersuchung als Ausweg/ When no-one knows how to continue-A representative socio-psychological aircraft noise study as a solution. *Zeitschrift für Lärmbekämpfung, 50*(5).
30. Moudon, A. V. (2009). Real noise from the urban environment: how ambient community noise affects health and what can be done about it. *American Journal of Preventive Medicine, 37*(2), 167-171. doi: 10.1016/j.amepre.2009.03.019.
31. Murillo, I. C. (2007). How does noise affect us? In our health, life styles and environs. *Revista de enfermeria (Barcelona, Spain)*, *30*(10), 13-6.
32. Kryter, K. D. & Grandjean, E. (1960): *Mensch* & *Umwelt*. Die *Wirkungen des Lärms auf den Menschen.* Verlag: J. R. Geigy, Basel
33. Ortscheid, J., & Wende, H. (2000). Fluglärmwirkungen, Umweltbundesamt.
34. Pichot, P. (1992). Noise, sleep and behavior. *Bulletin de l'Academie Nationale de Médecine*, *176*(3), 393-9.

35. Pirrera, S., De Valck, E., & Cluydts, R. (2010). Nocturnal road traffic noise: A review on its assessment and consequences on sleep and health. *Environment International*, *36*(5), 492-498. doi: 10.1016/j.envint.2010.03.007
36. Röösli, M. (2013). Health effects of environmental noise exposure. *Therapeutische Umschau. Revue Therapeutique*, *70*(12), 720-724. doi: 10.1024/0040-5930/a000470
37. Schick, A., & Meis, M. (2001). Reducing individual noise-induced health risks with health psychology and stress coping. *Schriftenreihe des Vereins für Wasser-, Boden-und Lufthygiene*, (111), 106.
38. Schust, M. (2004). Effects of low frequency noise up to 100 Hz. *Noise and Health*, *6*(23), 73.
39. Shepherd, M. (1975). Pollution, noise, and mental health. *The Lancet*, *305*(7902), 322-324. doi: 10.1016/S0140-6736(75)91222-2
40. Spooner, H. J. (1922). Health problems involved in noise and fatigue. *Nation's Health*, *4*(91-95), 156-159
41. Spreng, M. (2004). Noise induced nocturnal cortisol secretion and tolerable overhead flights. *Noise and Health*, *6*(22), 35.
42. Stansfeld, S. A., Haines, M. M., Burr, M., Berry, B., & Lercher, P. (2000). A review of environmental noise and mental health. *Noise and Health*, *2*(8), 1.
43. Stansfeld, S. A. & Clark, C. (2008). Noise and psychiatric disorder. In: The impact of the Environment on Psychiatric Disorder (Freeman, H. & Stansfeld, S. eds), Routledge, Taylor & Francis Group. London & New York
44. Stansfeld, S., & Clark, C. (2015). Health effects of noise exposure in children. *Current Environmental Health Reports*, *2*(2), 171-178. doi: 10.1007/s40572-015-0044-1
45. Talbott, E., & Thompson, S. J. (1995). Health Effects from Environmental Noise. In: An Introduction to Environmental Epidemiology (Talbott, E. & Craun, G. F. eds.), Lewis Publishers. Boca Ranton, New York, London & Tokyo. 209.
46. Tarnopolski, A. (1979). The effects of aircraft noise. *Journal of Psychosomatic Research*, 23(6), 371-372.
47. Thompson, S. (1996). Non-auditory healty effects of noise: updated review. *Noise Control-The Next 25 Years, Proceedings of the Internoise'96, Institute of Acoustics*, 2177-2182.
48. Van Kamp, I., & Davies, H. (2008). Environmental noise and mental health: Five year review and future directions. In *Proceedings of the 9th International Congress on Noise as a Public Health Problem*.
49. van Kamp, I., van Kempen, E., Baliatsas, C., & Houthuijs, D. (2013). Mental health as context rather than health outcome of noise: competing hypotheses regarding the role of sensitivity, perceived soundscapes and restoration. In *Proceedings Internoise*.
50. Ward, W D. (1988). Jet noise and mental health. *British Journal of Audiology,* 22(2), 157-158.
51. Yano, T., Gjestland, T., & Lee, S. (2012). Community response to noise. *Noise and Health*, *14*(61), 303. doi: 10.4103/1463-1741.104898

Exposure (not noise, or noise not measured or modelled) (n = 40)

1. Abey-Wickrama, I., a'Brook, M. F., Gattoni, F. E. G., & Herridge, C. F. (1969). Mental-hospital admissions and aircraft noise. *The Lancet*, *294*(7633), 1275-1277. doi: 10.1016/S0140-6736(69)90810-1
2. Chen, H., Kwong, J. C., Copes, R., Tu, K., Villeneuve, P. J., Van Donkelaar, A., ... & Wilton, A. S. (2017). Living near major roads and the incidence of dementia,

Parkinson's disease, and multiple sclerosis: a population-based cohort study. *The Lancet*, *389*(10070), 718-726. doi:10.1016/S0140-6736(16)32399-6

3. Devroey, D., Betz, W., & Coigniez, P. (2002). Influence of noise on the patients, health perception: An epidemiological registration. *Tijdschrift voor Geneeskunde*, *58*(21), 1392-7.
4. Dreger, S., Meyer, N., Fromme, H., & Bolte, G. (2015). Environmental noise and incident mental health problems: A prospective cohort study among school children in Germany. *Environmental Research*, *143*, 49-54. doi: 10.1016/j.envres.2015.08.003
5. Dzhambov, A. M., Markevych, I., Tilov, B., Arabadzhiev, Z., Stoyanov, D., Gatseva, P., & Dimitrova, D. D. (2018). Pathways linking residential noise and air pollution to mental ill-health in young adults. *Environmental Research*, *166*, 458-465. doi: 10.1016/j.envres.2018.06.031
6. Firdaus, G., & Ahmad, A. (2010). Noise pollution and human health: a case study of municipal corporation of Delhi. *Indoor and Built Environment*, *19*(6), 648-656. doi: 10.1177/1420326X10370532
7. Francois, J. (1981). Effect of aircraft noise on the equilibrium of airport residents: Longitudinal study around Roissy, phase 3.
8. Fyhri, A., & Klæboe, R. (2009). Road traffic noise, sensitivity, annoyance and self-reported health—A structural equation model exercise. *Environment International*, *35*(1), 91-97. doi: 10.1016/j.envint.2008.08.006
9. Gattoni, F., & Tarnopolsky, A. (1973). Aircraft noise and psychiatric morbidity. *Psychological Medicine*, *3*(4), 516-520. doi: 10.1017/S0033291700054337
10. Gielkens C, Roberts A. Geluidshinder en de (ervaren) gezondheid: het vervolg. geluid. 2003;1:9–12.
11. Hand, D. J., Tarnopolsky, A., Barker, S. A., & Jenkins, L. M. (1980). Relationships between psychiatric hospital admissions and aircraft noise: A new study. *Proc. Int. Congr. Noise as a Public Health Problem, ASHA Rep*, *10*, 277-282.
12. Hardoy, M. C., Carta, M. G., Marci, A. R., Carbone, F., Cadeddu, M., Kovess, V., ... & Carpiniello, B. (2005). Exposure to aircraft noise and risk of psychiatric disorders: the Elmas survey. *Social Psychiatry and Psychiatric Epidemiology*, *40*(1), 24-26. doi:10.1007/s00127-005-0837-x
13. Hatfield, J., Job, R. F. S., Carter, N. L., Peploe, P., Taylor, R., & Morrell, S. (2001). The influence of psychological factors on self-reported physiological effects of noise. *Noise and Health*, *3*(10), 1.
14. Hatfield, J., Job, R. S., Hede, A. J., Carter, N. L., Peploe, P., Taylor, R., & Morrell, S. (2002). Human response to environmental noise: the role of perceived control. *International Journal of Behavioral Medicine*, *9*(4), 341-359. doi: 10.1207/S15327558IJBM0904_04
15. Hattori, M. (2000). A field study of health effects of aircraft noise in adults around Komatsu Air Base (1998). *[Nihon koshu eisei zasshi] Japanese Journal of Public Health*, *47*(1), 20-31.
16. Herridge, C. F. (1972). Aircraft noise and mental hospital admission. *British Journal of Audiology*, *6*(2), 32-36. doi: 10.3109/00381797209075555
17. Jenkins, L. M., Tarnopolsky, A., Hand, D. J., & Barker, S. M. (1979). Comparison of three studies of aircraft noise and psychiatric hospital admissions conducted in the same area. *Psychological Medicine*, *9*(4), 681-693. doi: 10.1017/S0033291700033997
18. Jenkins, L., Tarnopolsky, A., & Hand, D. (1981). Psychiatric admissions and aircraft

noise from London Airport: four-year, three-hospitals' study. *Psychological Medicine*, *11*(4), 765-782. doi: 10.1017/S0033291700041271

19. Jensen, H. A., Rasmussen, B., & Ekholm, O. (2018). Neighbour and traffic noise annoyance: a nationwide study of associated mental health and perceived stress. *European Journal of Public Health*, *28*(6), 1050-1055. doi: 10.1093/eurpub/cky091.
20. Kishikawa, H., Matsui, T., Uchiyama, I., Miyakawa, M., Hiramatsu, K., & Stansfeld, S. A. (2009). Noise sensitivity and subjective health: Questionnaire study conducted along trunk roads in Kusatsu, Japan. *Noise and Health*, *11*(43), 111. doi: 10.4103/1463-1741.50696
21. Knipschild, P. (1977). V. Medical effects of aircraft noise: community cardiovascular survey. *International Archives of Occupational and Environmental Health*, *40*(3), 185-190. doi: 10.1007/BF01842081
22. Knipschild, P. (1977). VI. Medical effects of aircraft noise: general practice survey. *International Archives of Occupational and Environmental Health*, *40*(3), 191-196. doi: 10.1007/BF01842082
23. Knipschild, P., & Oudshoorn, N. (1977). VII. Medical effects of aircraft noise: drug survey. *International Archives of Occupational and Environmental Health*, *40*(3), 197-200. doi: 10.1007/BF01842083
24. Koszarny, Z., Szata, W., & Goryński, P. (1982). Comparative evaluation of indicators of street noise with reference to the effect on the Warsaw's population. *Roczniki Panstwowego Zakladu Higieny*, *33*(5-6), 469-481.
25. Koszarny, Z. (2000). The effect of intensive traffic noise on well-being and self-assessed health status of urban population. *Roczniki Panstwowego Zakladu Higieny*, *51*(2), 191-201.
26. Kryter, K. D. (1990). Aircraft noise and social factors in psychiatric hospital admission rates: a re-examination of some data. *Psychological Medicine*, *20*(2), 395-411. doi: 10.1017/S0033291700017712
27. Kryter, K. D. (1990). "Aircraft noise and social factors in psychiatric hospital admission rates: A re-examination of some data": Erratum.
28. Lee, K. W., Kim, S. J., Park, J. B., Min, K. B., Kil, H. G., Lee, C., & Lee, K. J. (2011). Relationship Between Aircraft Noise Exposure and Depression, Anxiety, Stress in Korea. *Epidemiology*, *22*(1), S258.
29. Miyakawa, M., Matsui, T., Uchiyama, I., Hiramatsu, K., Hayashi, N., Morita, I., ... & Ohashi, S. (2008). Relationship between subjective health and disturbances of daily life due to aircraft noise exposure—Questionnaire study conducted around Narita International Airport—. In *Proc. 9th International conference on Noise as a Public Health Problem* (pp. 314-321).
30. Niemann, H., Maschke, C., & Hecht, K. (2005). Noise induced annoyance and morbidity. Results from the pan European LARES-survey. *Bundesgesundheitsblatt, Gesundheitsforschung, Gesundheitsschutz*, *48*(3), 315-328. doi: 10.4103/1463-1741.33537
31. Niemann, H., Bonnefoy, X., Braubach, M., Hecht, K., Maschke, C., Rodrigues, C., & Robbel, N. (2006). Noise-induced annoyance and morbidity results from the pan-European LARES study. *Noise and Health*, *8*(31), 63. doi: 10.4103/1463-1741.33537
32. Pisani, S., Bonarrigo, D., Gambino, M., Macchi, L., Banfi, F., Verri, A. M., ... & Cortinovis, I. (2003). Salus domestica epidemiological study: evaluation of health harm in a sample of women living close to Malpensa 2000 Airport. *EPIDEMIOLOGIA E PREVENZIONE*, (4), 234-241.

33. Putrik, P., de Vries, N. K., Mujakovic, S., van Amelsvoort, L., Kant, I., Kunst, A. E., ... & Jansen, M. (2015). Living environment matters: relationships between neighborhood characteristics and health of the residents in a Dutch municipality. *Journal of Community Health*, *40*(1), 47-56. doi: 10.1007/s10900-014-9894-y
34. Ristovska, G., Gjorgjev, D., & Jordanova, N. P. (2004). Psychosocial effects of community noise: cross sectional study of school children in urban center of Skopje, Macedonia. *Croatian Medical Journal*, *45*(4), 473-476.
35. Tarnopolsky, A., Barker, S. M., Wiggins, R. D., & McLean, E. K. (1978). The effect of aircraft noise on the mental health of a community sample: a pilot study. *Psychological Medicine*, *8*(2), 219-233. doi: 10.1017/S0033291700014276
36. Tarnopolsky, A., Watkins, G., & Hand, D. J. (1980). Aircraft noise and mental health: I. Prevalence of individual symptoms. *Psychological Medicine*, *10*(4), 683-698. doi: 10.1017/S0033291700054982
37. Tarnopolsky, A., Morton-Williams, J., & Barker, S. M. (1980). *Aircraft noise and prevalence of psychiatric disorders*. Social & Community Planning Research.
38. Turnovska, T., Staykova, J., & Petkov, T. (2004). Health assessment of populations living close to the airport of Bourgas, Bulgaria. *Arhiv za Higijenu rada i Toksikologiju*, *55*(1), 5-10.
39. Watkins, G., Tarnopolsky, A., & Jenkins, L. M. (1981). Aircraft noise and mental health: II. Use of medicines and health care services. *Psychological Medicine*, *11*(1), 155-168 doi: 10.1017/S003329170005337X.
40. Weigl, K., Herr, C. E., Meyer, N., Otto, C., Stilianakis, N., Bolte, G., ... & Kolb, S. (2018). Predictors of Health-related Quality of Life in Bavarian Preschool Children. *Gesundheitswesen*, *80* (S 01): S1-S4. doi: 10.1055/s-0042-104117

Unrelated research topic (n = 12)

1. Agaeva, M. (2010). Precedence effect for moving sound. *International Journal of Psychophysiology*, *77*(3), 302. doi: 10.1016/j.ijpsycho.2010.06.195
2. Baliatsas, C., van Kamp, I., Swart, W., Hooiveld, M., & Yzermans, J. (2016). Noise sensitivity: Symptoms, health status, illness behavior and co-occurring environmental sensitivities. *Environmental Research*, *150*, 8-13. doi: 10.1016/j.envres.2016.05.029
3. Cantrell, R. W. (1974). Prolonged exposure to intermittent noise: audiometric, biochemical, motor, psychological and sleep effects. *The Laryngoscope*, *84* (10 Pt 2 Suppl 1), 1.
4. Favre, B. (1985). Relationship between traffic noise and effect in people; development of an exposure model. *Rev Acoust.* 18(74), 409-421.
5. Favre, B. (1985). Relationship between traffic noise and effect in people; application to a typical urban environment. *Rev Acoust.* 18(74), 423-438.
6. Hayashi, C., Hayashi, F., Kodama, H., & Kondo, S. (1973). Construction of PAANI (Psychological Assessment of Aircraft Noise Index) using statistical and psychological ideas. *Proceedings of the Institute of Statistical Mathematics, 1*(21).
7. Kryter, K. D. (2007). Acoustical, sensory, and psychological research data and procedures for their use in predicting effects of environmental noises. *The Journal of the Acoustical Society of America*, *122*(5), 2601-2614. doi: 10.1121/1.2782748
8. Lercher, P., Schmitzberger, R., & Kofler, W. (1995). Perceived traffic air pollution, associated behavior and health in an alpine area. *Science of the Total Environment*, *169*(1-3), 71-74. doi: 10.1016/0048-9697(95)04634-D

9. Stansfeld, S. A., Clark, C. R., Jenkins, L. M., & Tarnopolsky, A. (1985). Sensitivity to noise in a community sample: I. Measurement of psychiatric disorder and personality. *Psychological Medicine*, *15*(2), 243-254. doi: 10.1017/S0033291700023527
10. Stansfeld, S. A., Gallacher, J. E. J., Sharp, D. S., & Yarnell, J. W. G. (1991). Social factors and minor psychiatric disorder in middle-aged men: a validation study and a population survey. *Psychological Medicine*, *21*(1), 157-167. doi: 10.1017/S0033291700014744
11. Stansfeld, S. A. (1992). Noise, noise sensitivity and psychiatric disorder: epidemiological and psychophysiological studies. *Psychological Medicine Monograph Supplement*, *22*, 1-44. doi: 10.1017/S0264180100001119
12. Wunderli, J. M., Pieren, R., Habermacher, M., Vienneau, D., Cajochen, C., Probst-Hensch, N., ... & Brink, M. (2016). Intermittency ratio: A metric reflecting short-term temporal variations of transportation noise exposure. *Journal of Exposure Science and Environmental Epidemiology*, *26*(6), 575. doi:10.1038/jes.2015.5

Full-Text could not be obtained (n = 9)

1. Albertini, C. (1931). La lotta contro i rumori ei rumori della strada. Vita Italiana
2. Guthof, O., & Gableske, R. (1968). Effects of traffic noise on human life. *Das Öffentliche Gesundheitswesen*, *30*(1), 1-6.
3. Haider, M. (1976). Air pollution, noise-current environmental problems. *Wiener Medizinische Wochenschrift (1946)*, *126*(6), 65.
4. Ising, H. & Rebentisch, E. (1992). Environmental noise: Exposure, annoyance and long-term effects on health. [German]. *Wissenschaft und Umwelt*, (2), 147-150.
5. Kelly, A. (1986). Effects of aircraft noise and noise sensitivity on the physical and mental well-being of older adults. Keine weiteren Angaben vorhanden.
6. Lohman, A. (2007). The impact of a freeway on neighborhood: Sense of community, size, and methods of measurement. Dissertation. The Claremont Graduate University. AAI3233758.
7. Schick, A. (1992): The psychological aspects of noise research. *Zeitschrift für Lärmbekämpfung*, 39 (4) 113-117.
8. Vartiainen, A.-K., Turunen, A. W., Ung-Lanki, S. & Lanki, T. (2015). Meluherkkyydellä on tärkeä rooli melun kokemisessa. *Psykologia, 50(4),* 244-256.
9. Yamamoto, K. (1993). Living environment stress and mental health--From the ten years' study of psychosocial stress in inhabitants along the loop road 7 in Metropolitan Tokyo. *Journal of Mental Health*, 39, 41-54.

Screening instrument was not validated (n = 6)

1. Belojević, G., Jakovljević, B., & Aleksić, O. (1997). Subjective reactions to traffic noise with regard to some personality traits. *Environment International*, *23*(2), 221-226. doi: 10.1016/S0160-4120(97)00008-1
2. Öhrström, E. (1989). Sleep disturbance, psycho-social and medical symptoms—a pilot survey among persons exposed to high levels of road traffic noise. *Journal of Sound and Vibration*, *133*(1), 117- doi: 10.1016/0022-460X(89)90986-3
3. Öhrström, E. (1991). Psycho-social effects of traffic noise exposure. *Journal of Sound and Vibration*, *151*(3), 513-517. doi: 10.1016/0022-460X(91)90551-T
4. Öhrström, E., & Skånberg, A. (2000). Adverse health effects in relation to noise mitigation—A longitudinal study in the city of Göteborg. In *Proceedings of the 29th International Congress and Exhibition on Noise Control Engineering, Nice, France* (pp. 27-30).

5. Öhrström, E. (2004). Longitudinal surveys on effects of changes in road traffic noise—annoyance, activity disturbances, and psycho-social well-being. *The Journal of the Acoustical Society of America*, *115*(2), 719-729. doi: 10.1121/1.1639333
6. Yoshida, T., Osada, Y., Kawaguchi, T., Hoshiyama, Y., Yoshida, K., & Yamamoto, K. (1997). Effects of road traffic noise on inhabitants of Tokyo. *Journal of Sound and Vibration*, *205*(4), 517-522. doi: 10.1006/jsvi.1997.1020

Industry-/ Military Aircraft Noise (n = 4)

1. Hiramatsu, K., Yamamoto, T., Taira, K., Ito, A., & Nakasone, T. (1997). A survey on health effects due to aircraft noise on residents living around Kadena air base in the Ryukyus. *Journal of Sound and Vibration*, *205*(4), 451-460. doi: 10.1006/jsvi.1997.1011
2. Hiramatsu, K., Matsui, T., Miyakita, T., Ito, A., Tokuyama, T., Osada, Y., & Yamamoto, T. (2002). Population-based questionnaire survey on health effects of aircraft noise on residents living around US airfields in the Ryukyus—Part II: An analysis of the discriminant score and the factor score. *Journal of Sound and Vibration*, *250*(1), 139-144. doi: 10.1006/jsvi.2001.3896
3. Matsui, T. (2013, September). Psychosomatic disorder due to aircraft noise and its causal pathway. In *INTER-NOISE and NOISE-CON Congress and Conference Proceedings, 247*(8), 605-609. Institute of Noise Control Engineering.
4. Stošić, L., & Blagojević, L. (2011). Environmental noise and mental disturbances in urban population. *Acta Medica Medianae*, *50*(3), 34-39. doi: 10.5633/amm.2011.0306

Population of employees considered (not general population) (n = 3)

1. Akan, Z., Yilmaz, A., Özdemir, O., & Korpinar, M. A. (2012). Noise pollution, psychiatric symptoms and quality of life: noise problem in the east region of Turkey. *Journal of Inonu University Medical Faculty*, *19*(2), 75-81. doi: 10.7247/jiumf.19.2.3
2. Calapaj, G. G., & Bellia, G. (1969). Psychometric studies of subjects exposed to the noise of jet engines. *La Medicina del Lavoro*, *60*(1), 43.
3. Chiovenda, P., Pasqualetti, P., Zappasodi, F., Ercolani, M., Milazzo, D., Tomei, G., ... & Tecchio, F. (2007). Environmental noise-exposed workers: Event-related potentials, neuropsychological and mood assessment. *International Journal of Psychophysiology*, *65*(3), 228-237. doi:10.1016/j.ijpsycho.2007.04.009

Experimental study design (n = 3)

1. Naqvi, F., Haider, S., Perveen, T., & Haleem, D. J. (2012). Sub-chronic exposure to noise affects locomotor activity and produces anxiogenic and depressive like behavior in rats. *Pharmacological Reports*, *64*(1), 64-69. doi: 10.1016/S1734-1140(12)70731-4
2. Granati, *A.,* Angeleri, F., Lenzi, R. (1959). *L'*influenza dei rumori sul sistema nervoso – Folia Medica: XLII (11): 1314-1325.
3. Schönpflug, W., Kausche, J. & Wieland, R. (1978). Traffic noise during leisure time. [German]. *Kampf dem Lärm*, 25(1): 21-25.

Results not usable (see reference for more details) (n = 3)

1. Bodin, T., Albin, M., & Bjork, J. (2013). Road traffic noise and mental health-Preliminary results from a cross-sectional study in southern Sweden. In *INTER-NOISE and NOISE-CON Congress and Conference Proceedings*, 247(2), 5768-5772. Institute of Noise Control Engineering.

   Only descriptive results depicted without numbers;
   Author did not respond to requests for more information
2. Greiser, E., Greiser, C., & Janhsen, K. (2007). Night-time aircraft noise increases

prevalence of prescriptions of antihypertensive and cardiovascular drugs irrespective of social class—the Cologne-Bonn Airport study. *Journal of Public Health, 15*(5), 327-337. doi: 10.1007/s10389-007-0137-x

Anxiolytics were evaluated together with blood pressure and heart medications

3. Houthujis, D. J. M. & van Wiechen, C. M. A. G. (2006). Monitoring van gezondheid en beleving rondom de luchthaven Schiphol. RIVM rapport 630100003/2006

   Author did not respond to requests for more information

Convenience Sample (n = 2)

1. Dzhambov, A., Tilov, B., Markevych, I., & Dimitrova, D. (2017). Residential road traffic noise and general mental health in youth: the role of noise annoyance, neighborhood restorative quality, physical activity, and social cohesion as potential mediators. *Environment International, 109*, 1-9. doi: 10.1016/j.envint.2017.09.009.
2. Maschke, C., & Hecht, K. (2005). Tag-Nacht Unterschiede in der multifaktoriellen Genese von lärminduzierten Erkrankungen–Ergebnisse einer epidemiologischen Studie. *Somnologie, 9*(2), 96-104. doi: 10.1111/j.1439-054X.2005.00046.x

Repeat publication (n = 1)

1. Greiser, E., & Glaeske, G. (2013). Social and economic consequences of night-time aircraft noise in the vicinity of Frankfurt/Main airport. *Gesundheitswesen (Bundesverband der Ärzte des Öffentlichen Gesundheitsdienstes (Germany)), 75*(3), 127-133.

Outcome not differentiated (n = 1)

1. Wright, D. M., Newell, K., Maguire, A., & O'Reilly, D. (2018). Aircraft noise and self-assessed mental health around a regional urban airport: a population based record linkage study. *Environmental Health, 17*(1), 74. doi: 10.1186/s12940-018-0418-6.

Table S2. Detailed extraction table of 8 studies (10 publications)

**Reference** (First author, publication year)<br><br>**Meta-analysis** (Yes/No)	**Study design**	**Population**	**Outcome**	**Exposure**			**Results**	**Comments** (study quality [overall assessment according to SIGN/CASP], conflict of interest [stated vs. not stated], funding [financed from public funds vs. financed from industry], confounding, strengths / weaknesses [potential bias, over- or underestimation of potential effects])
		**Study region**<br>**Sample population**<br>**Sample size** (M, F, M+F):<br>**Age** (mean, range)<br>**No. of cases / no. of controls or exposed/unexposed**<br>**Time of recruitment / follow-up** (mean, range)<br>**Response (%)**<br>(baseline minus loss to follow-up)	**Disease (ICD-10)**<br>**Prescription**<br>**Questionnaire**	**exposure source**	**exposure assessment**	**exposure levels**		
**Belojevic 2012**<br><br>**No**	Cross-sectional	**Study region:**<br>Belgrade, Serbia<br><br>**Sample size**:<br>$N_{Schools}$ =8<br>$N_{children}$= ca. 2000 -> 1150 -> 311<br>M=146<br>F=165<br>$N_{teacher}$= 102 -> 77<br><br>**Sample population:**<br>School children in Belgrade<br><br>**Age:**<br>7-11 years<br><br>**Time of recruitment:**<br>September 2008-June 2009	Adapted version of the Attention Deficit Disorder Questionnaire (5 item scale) completed by teachers to measure Executive Functioning (EF)	Road traffic noise<br>(1) children's home: middle of 115 municipal streets in September five (15 min.) measurements per day (8-10 am, 2-4 pm, 6-8 pm and 10-12 pm and midnight -	(1) calculated L_{24h} for each street	home: 40-80 dB	Table 5: Linear regression with executive functioning as dependent variable and predictors: gender, socioeconomic status and 24h-h equivalent noise level at home<br>Multivariate Model 2:<br>**Beta= -0.09**<br>**95% CI -0.17 to -0.01**<br><br>(Model 3: interaction noise x gender included, P=0,03)	**Study quality**<br>**-**<br><br>**Conflict of interest**<br>No information<br><br>**Funding**<br>Serbian Ministry of Science and Technological Development, Contract No. 175 078. (public funding)<br><br>**Confounding**<br>Gender, socioeconomic status<br><br>**Strengths/**

Reference (First author, publication year)<br><br>**Meta-analysis** (Yes/No)	**Study design**	**Population**	**Outcome**	**Exposure**			**Results**	**Comments** (study quality [overall assessment according to SIGN/CASP], conflict of interest [stated vs. not stated], funding [financed from public funds vs. financed from industry], confounding, strengths / weaknesses [potential bias, over- or underestimation of potential effects])
		**Study region**<br>**Sample population**<br>**Sample size** (M, F, M+F):<br>**Age** (mean, range)<br>**No. of cases / no. of controls or exposed/unexposed**<br>**Time of recruitment / follow-up** (mean, range)<br>**Response (%)**<br>(baseline minus loss to follow-up)	**Disease (ICD-10)**<br>**Prescription**<br>**Questionnaire**	**exposure source**	**exposure assessment**	**exposure levels**		
		**Response:**<br>57.5% (parental permission)<br>75.5% teacher participation		2 am)<br><br>(2) schools (in front): three daytime intervals (9-11 am, 12-2 pm and 3-5 pm)				**weaknesses:**<br>+ ethical clearance<br>- no common exposure measurement<br>- cross-sectional design<br>- no adequate control of cofounders<br>(-) noise exposure at school not considered in multivariate analysis, no correlation between school noise and EF
**Clark 2013**<br><br>**No** (continuous outcome, linear	longitudinal cohort (follow-up) study	**Study region:** West London, United Kingdom<br><br>**Sample size**:<br>29 schools<br>Eligible sample:<br>M+F=1,015,<br>Participated:	Mental health according to Strengths and Difficulties Questionnaire (SDQ)<br>- Hyperactivity<br>- Conduct	Aircraft<br>Road traffic	Aircraft:<br>$L_{eq,16h}$<br>16-h outdoor dB at school (07-23h).<br><br>Baseline data:<br>July to September	Analysis with continuous noise data<br><br>Cumulative	<u>Regression coefficient (β) for a 1 dB increase in noise exposure to a) aircraft noise at primary school, b) aircraft noise at secondary school, and c) cumulative aircraft noise exposure on cognition and health outcomes at follow-up (N = 461), (from Table 4)</u>	**Study quality:**<br>- (to +)<br><br>**Conflict of interest:**<br>not stated<br><br>**Funding:**<br>not stated

Reference (First author, publication year)<br><br>**Meta-analysis** (Yes/No)	Study design	Population	Outcome	Exposure			Results	Comments<br>(study quality [overall assessment according to SIGN/CASP], conflict of interest [stated vs. not stated], funding [financed from public funds vs. financed from industry], confounding, strengths / weaknesses [potential bias, over- or underestimation of potential effects])
		**Study region**<br>**Sample population**<br>**Sample size** (M, F, M+F):<br>**Age** (mean, range)<br>**No. of cases / no. of controls or exposed/unexposed**<br>**Time of recruitment / follow-up** (mean, range)<br>**Response (%)**<br>(baseline minus loss to follow-up)	**Disease (ICD-10)**<br>**Prescription**<br>**Questionnaire**	**exposure source**	**exposure assessment**	**exposure levels**		
regression)		M+F=461<br>M=202, F=259<br><br>**Sample population:**<br>RANCH study<br>At baseline, 9-10 year old children in schools around Heathrow airport in West London, followed up to secondary schools<br><br>**Age:**<br>range<br>15years,5months-17years,7months<br><br>**Exposed/unexposed:**<br>not applicable<br><br>**Time of recruitment/ follow-up:**<br>2002-2003 / follow-up 2008<br><br>**Response:**	Problems<br>- Emotional symptoms<br>- Pro-social behavior<br>- Peer problems<br>- Total psychological distress score (scales added together, excluding pro-social behavior)		1999;<br>Follow up: July to September 2007.<br><br>(Road traffic only at baseline)	aircraft noise = mean of aircraft noise exposure at primary and secondary schools	(1) Longitudinal = Main analysis<br>(2) Cross-sectional<br>(3) Longitudinal<br><br>**Psychological distress at follow-up**<br>(1) Aircraft noise primary schools β = 0.006, 95% CI (-0.022, 0.061), p =0.998<br>(2) Aircraft noise secondary schools β = 0.017, 95% CI (-0.101, 0.135), p =0.781<br>(3) Cumulative aircraft noise at school β = 0.015, 95% CI (-0.069, 0.100), p =0.718<br><br>**Hyperactivity at follow-up**<br>(1) Aircraft noise primary schools β = 0.001, 95% CI (-0.060, 0.033), p =0.688<br>(2) Aircraft noise secondary schools β = 0.019, 95% CI (-0.034, 0.073), p =0.476<br>(3) Cumulative aircraft noise at school	**Confounding (adjusted for):**<br>age, gender, parental employment, crowding in the home, home ownership, mother's education, long standing illness, main language spoken at home, parental support, classroom glazing, and road noise at primary school.<br><br>**Strengths/ weaknesses:**<br>+longitudinal study<br>+ Participant-Non-participant analysis<br>+ adequate confounder assessment (but air pollution is missing)<br>- Loss-to follow-up: 55%

Reference (First author, publication year) **Meta-analysis** (Yes/No)	**Study design**	**Population**	**Outcome**	**Exposure**			**Results**	**Comments** (study quality [overall assessment according to SIGN/CASP], conflict of interest [stated vs. not stated], funding [financed from public funds vs. financed from industry], confounding, strengths / weaknesses [potential bias, over- or underestimation of potential effects])
		**Study region**<br>**Sample population**<br>**Sample size** (M, F, M+F):<br>**Age** (mean, range)<br>**No. of cases / no. of controls or exposed/unexposed**<br>**Time of recruitment / follow-up** (mean, range)<br>**Response (%)**<br>(baseline minus loss to follow-up)	**Disease (ICD-10)**<br>**Prescription**<br>**Questionnaire**	**exposure source**	**exposure assessment**	**exposure levels**		
		Baseline: no information<br><br>**Loss-to-follow up**:<br>55%					β = 0.010, 95% CI (-0.029, 0.002), p =0.613)<br><br>**Conduct problems at follow-up**<br>(1) Aircraft noise primary schools β = 0.006, 95% CI (-0.017, 0.029), p =0.616<br>(2) Aircraft noise secondary schools β = 0.015, 95% CI (-0.031, 0.060), p =0.527<br>(3) Cumulative aircraft noise at school β = 0.008, 95% CI (-0.024, 0.041), p =0.617<br><br>**Emotional symptoms at follow-up**<br>(1) Aircraft noise primary schools β = - 0.008, 95% CI (-0.035, 0.019), p =0.555<br>(2) Aircraft noise secondary schools β = -0.022, 95% CI (-0.073, 0.029), p =0.394<br>(3) Cumulative aircraft noise at school β = -0.015, 95% CI (-0.054, 0.023), p	- funding and conflict of interest not stated

Reference (First author, publication year)<br><br>Meta-analysis (Yes/No)	Study design	Population	Outcome	Exposure			Results	Comments (study quality [overall assessment according to SIGN/CASP], conflict of interest [stated vs. not stated], funding [financed from public funds vs. financed from industry], confounding, strengths / weaknesses [potential bias, over- or underestimation of potential effects])
		**Study region**<br>**Sample population**<br>**Sample size** (M, F, M+F):<br>**Age** (mean, range)<br>**No. of cases / no. of controls or exposed/unexposed**<br>**Time of recruitment / follow-up** (mean, range)<br>**Response (%)**<br>(baseline minus loss to follow-up)	**Disease (ICD-10)**<br>**Prescription**<br>**Questionnaire**	**exposure source**	**exposure assessment**	**exposure levels**		
							=0.436	
**Crombie 2011 (together with Stansfeld 2005 & Clark 2012)**<br><br>**No** (continuous outcome, linear regression)	Cross-sectional study	**Study region**<br>Heathrow Airport (London, UK), Barajas Airport (Madrid, Spain), Schiphol Airport (Amstedam, The Netherlands)<br><br>**Sample population:**<br>RANCH project: Students from schools around major airports in three European countries<br><br>**Sample size:**<br>M+F= 1,900<br>M=897, F=1,003<br><br>**Age**<br>Mean: 10.6 years<br><br>**Exposed/unexposed:**<br>For each of the 16 noise exposure	Mental health according to the Strengths and Difficulties Questionnaire (SDQ):<br>- Hyperactivity<br>- Conduct Problems<br>- Emotional symptoms<br>- Total score	Aircraft<br>Road traffic	**Aircraft noise:**<br>$L_{eq,16}$ (07h-23h)<br><br>**Road traffic noise:**<br>- The Netherlands: Modelled data<br>- UK, Spain: combination of modelling the proximity to motorways, major roads, and minor roads, traffic flow data, noise measurements taken at façade of school building	Continuous analysis<br>β per 1-dB increase in aircraft noise<br><br>Aircraft noise<br>Range: 30-77 dB<br><br>Road traffic noise<br>Range: 32-71 dB	Effect of aircraft noise on the SDQ measures (from Table 3):<br>**Hyperactivity:**<br>Model 2:<br>Aircraft noise: β= 0.01, 95% CI: 0.00-0.02, **p=0.05**<br>Model 3:<br>Aircraft noise: β= 0.01, 95% CI: 0.00-0.02, **p=0.05**<br><br>**Emotional problems:**<br>Model 2:<br>Aircraft noise: β= 0.00, 95% CI: -0.01-0.01, p=0.34<br>Model 3:<br>Aircraft noise: β= 0.00, 95% CI: -0.01-0.01, p=0.97<br><br>**Conduct problems:**<br>Model 2:	**Study quality:**<br>-<br>Cross-sectional study design<br><br>**Conflict of interest:**<br>stated (The authors declare that they have no competing interest)<br><br>**Funding:**<br>stated (RANCH Study: European Community, UK co-funding by Department of Environment, Food and rural Affairs; Netherlands co-funding by Dutch Ministry of Public

Reference (First author, publication year)<br><br>**Meta-analysis** (Yes/No)	**Study design**	**Population**	**Outcome**	**Exposure**			**Results**	**Comments** (study quality [overall assessment according to SIGN/CASP], conflict of interest [stated vs. not stated], funding [financed from public funds vs. financed from industry], confounding, strengths / weaknesses [potential bias, over- or underestimation of potential effects])
		**Study region**<br>**Sample population**<br>**Sample size** (M, F, M+F):<br>**Age** (mean, range)<br>**No. of cases / no. of controls or exposed/unexposed**<br>**Time of recruitment / follow-up** (mean, range)<br>**Response (%)**<br>(baseline minus loss to follow-up)	**Disease (ICD-10)**<br>**Prescription**<br>**Questionnaire**	**exposure source**	**exposure assessment**	**exposure levels**		
		grid cells: 2 schools from Spain (M+F=559), 2 schools from UK (M+F=783), 1 school the Netherlands (M+F=558)<br><br>**Time of recruitment / follow-up**<br>April- October 2002 (Stansfeld, 2005)<br><br>**Response**<br>89%					Aircraft noise: β= -0.01, 95% CI: -0.01-0.00, p=0.23<br>Model 3:<br>Aircraft noise: β= -0.01, 95% CI: -0.01-0.00, p=0.17<br><br>Model 2: adjusted for all confounding factors<br>Model 3: same as Model 3, but with addition of early biological risk as main effect<br><br><br>Table 3: Road traffic on the SDQ measures:<br>**Emotional problems:**<br>Model 2:<br>Road traffic: β= 0.00, 95% CI: -0.01-0.01, p=0.97<br>Model 3:<br>Road traffic: β= -0.00, 95% CI: -0.01-0.01, p=0.89<br><br>**Conduct problems:**	Health, Welfare, and Sports, Dutch Ministry of Spatial Planning, Housing and Environment and Dutch Ministry of Transport, Public Works and Water Management) → public funding<br><br>**Confounding (adjusted for):**<br>age, sex, country of origin, employment status, crowding at home, educational level of mother, housing tenancy, long-standing illnesses, main language spoken at home, parental support for school work, classroom

Reference (First author, publication year)<br><br>**Meta-analysis** (Yes/No)	Study design	Population	Outcome	Exposure			Results	Comments (study quality [overall assessment according to SIGN/CASP], conflict of interest [stated vs. not stated], funding [financed from public funds vs. financed from industry], confounding, strengths / weaknesses [potential bias, over- or underestimation of potential effects])
		**Study region**<br>**Sample population**<br>**Sample size** (M, F, M+F):<br>**Age** (mean, range)<br>**No. of cases / no. of controls or exposed/unexposed**<br>**Time of recruitment / follow-up** (mean, range)<br>**Response (%)**<br>(baseline minus loss to follow-up)	**Disease (ICD-10)**<br>**Prescription**<br>**Questionnaire**	**exposure source**	**exposure assessment**	**exposure levels**		
							Model 2:<br>Road traffic: β= -0.01, 95% CI: -0.02-0.00, p=0.04<br>Model 3:<br>Road traffic: β= -0.01, 95% CI: -0.02-0.00, p=0.03<br><br>**Hyperactivity:**<br>Model 2:<br>Road traffic: β= 0.00 (0.01), 95% CI: -0.01-0.01, p=0.96<br>Model 3:<br>Road traffic: β= -0.00 (0.01), 95% CI: -0.01-0.01, p=0.94<br><br>- Moderating effects of early biological risk assessed (dichotomous variable): information on child`s birth weight (<2500 g) and gestation period (<36 weeks)<br><br>**Mental health/ psychological distress:**<br>from Stansfeld 2005	glazing type<br><br>**Strengths/ weaknesses:**<br>+ high response (89%)<br>+ multiple noise sources considered<br>+ exposure assessment<br>+ adequate list of confounders<br>- air pollution not considered as confounder<br>- selection based on noise exposure at school → may have led to unrepresentative sample<br>- cross-sectional design

Reference (First author, publication year)<br><br>**Meta-analysis** (Yes/No)	**Study design**	**Population**	**Outcome**	**Exposure**			**Results**	**Comments** (study quality [overall assessment according to SIGN/CASP], conflict of interest [stated vs. not stated], funding [financed from public funds vs. financed from industry], confounding, strengths / weaknesses [potential bias, over- or underestimation of potential effects])
		**Study region**<br>**Sample population**<br>**Sample size** (M, F, M+F):<br>**Age** (mean, range)<br>**No. of cases / no. of controls or exposed/unexposed**<br>**Time of recruitment / follow-up** (mean, range)<br>**Response (%)**<br>(baseline minus loss to follow-up)	**Disease (ICD-10)**<br>**Prescription**<br>**Questionnaire**	**exposure source**	**exposure assessment**	**exposure levels**		
							Table 3: Cognitive and health outcomes and aircraft noise exposure<br>Model 1: β= 0.015, 95% CI: -0.012 to 0.042<br>Model 2: β= 0.013, 95% CI: -0.012 to 0.038 (fully adjusted)<br><br>Table 5: Cognitive and health outcomes and exposure to road traffic noise<br>Model 1: β= –0·012, 95% CI –0·045 to 0·021<br>Model 2: β= –0·018, 95% CI –0·049 to 0·013 (fully adjusted)<br><br>**Sensitivity analysis considering air pollution in a subset of children (n=634)** from Clark 2012<br><br>**SDQ total difficulties**<br>**Aircraft noise:**<br>adjusted model (table 2)<br>β =-0.023, 95% CI: -0.073 to 0.026<br>additionally adjusted for air pollution and road traffic noise (table 4):	

Reference (First author, publication year)<br><br>**Meta-analysis** (Yes/No)	**Study design**	**Population**<br>**Study region**<br>**Sample population**<br>**Sample size** (M, F, M+F):<br>**Age** (mean, range)<br>**No. of cases / no. of controls or exposed/unexposed**<br>**Time of recruitment / follow-up** (mean, range)<br>**Response (%)**<br>(baseline minus loss to follow-up)	**Outcome**<br>**Disease (ICD-10)**<br>**Prescription**<br>**Questionnaire**	**Exposure**<br>**exposure source**	**exposure assessment**	**exposure levels**	**Results**	**Comments**<br>(study quality [overall assessment according to SIGN/CASP], conflict of interest [stated vs. not stated], funding [financed from public funds vs. financed from industry], confounding, strengths / weaknesses [potential bias, over- or underestimation of potential effects])
							β= -0.028, 95% CI: -0.079 to 0.023<br><br>**Road noise:**<br>adjusted model (table 2)<br>β= -0.030, 95% CI: -0.093 to 0.033<br>additionally adjusted for air pollution and aircraft noise (table 4):<br>β= -0.037, 95% CI: -0.104 to 0.029	
**Forns 2016**<br><br>**No**	Cross-sectional<br><br>BREATHE project	**Study region:**<br>Barcelona, Spain<br><br>**Sample size**:<br>$N_{Schools}$=39<br>N= 2,897 (enrolled)<br>M=1,446<br>F=1,430<br><br>**Sample population:**<br>School children in Barcelona and Sant Cugat del Vallès (3 schools)<br><br>**Age:**	SDQ (parents)<br>ADHD-DSM-IV list (teacher)	Traffic noise in classroom<br><br>3x 10-Min. measurements taken for 2 consecutive days in a single classroom of each school in	L_{eq}<br>(average of two 30-min measurements)	28.8-51.1 dB indoor<br><br>Continuous analysis<br>(IQR = 7.60dB)	Table 4: SDQ total difficulties score and ADHD symptomatology<br><br>[ ] | Mean Ratios (per IQR) | 95% CI<br>Elementary carbon (EC) and noise (multiple exposure, indoor EC measurements)<br>SDQ | 0.98 | (0.92-1.04)<br>ADHD-Sympt | **1.29** | **1.18-1.43**<br>NO_2 and noise (multiple exposure, indoor NO_2 measurements)	**Study quality**<br>**-**<br><br>**Conflict of interest**<br>stated (none declared)<br><br>**Funding**<br>European Research Council (public funding)<br><br>**Confounding**<br>child's sex, child's age, maternal education,

Reference (First author, publication year)<br><br>**Meta-analysis** (Yes/No)	**Study design**	**Population**	**Outcome**	**Exposure**			**Results**	**Comments** (study quality [overall assessment according to SIGN/CASP], conflict of interest [stated vs. not stated], funding [financed from public funds vs. financed from industry], confounding, strengths / weaknesses [potential bias, over- or underestimation of potential effects])
		**Study region**<br>**Sample population**<br>**Sample size** (M, F, M+F):<br>**Age** (mean, range)<br>**No. of cases / no. of controls or exposed/unexposed**<br>**Time of recruitment / follow-up** (mean, range)<br>**Response (%)**<br>(baseline minus loss to follow-up)	**Disease (ICD-10)**<br>**Prescription**<br>**Questionnaire**	**exposure source**	**exposure assessment**	**exposure levels**		
		7-11 years<br><br>**Time of recruitment:**<br>2012-2013<br><br>**Response:**<br>59%		the morning before children arrived (before 0900 hours)<br><br>Air pollution (elemental carbon (EC), and NO_2)			SDQ 1.01 (0.94-1.07)<br>ADHD-Sympt **1.24** **(1.12-1.38)**<br><br>Multi-exposure models including traffic-related air pollutants (TRAPs) and noise were adjusted for child's sex, child's age, maternal education, urban vulnerability index at home address, air pollution (BC) at home, traffic noise annoyance at home, home tobacco use, urban vulnerability index at school, and type of school.	urban vulnerability index at home address, air pollution (BC) at home, traffic noise annoyance at home, home tobacco use, urban vulnerability index at school, and type of school and air pollution<br>**strengths/ weaknesses:**<br>+ air particulate level considered<br>+adequate control for confounders<br>+ ethical clearance<br>- no common exposure measurement and no exposure assessment descriptors<br>- cross-sectional design<br>(-) very brief noise

<table>
<tr><th rowspan="2">Reference (First author, publication year)

Meta-analysis (Yes/No)</th><th rowspan="2">Study design</th><th>Population</th><th>Outcome</th><th colspan="3">Exposure</th><th rowspan="2">Results</th><th rowspan="2">Comments
(study quality [overall assessment according to SIGN/CASP], conflict of interest [stated vs. not stated], funding [financed from public funds vs. financed from industry], confounding, strengths / weaknesses [potential bias, over- or underestimation of potential effects])</th></tr>
<tr><th>Study region
Sample population
Sample size (M, F, M+F):
Age (mean, range)
No. of cases / no. of controls or exposed/unexposed
Time of recruitment / follow-up (mean, range)
Response (%)
(baseline minus loss to follow-up)</th><th>Disease (ICD-10)
Prescription
Questionnaire</th><th>exposure source</th><th>exposure assessment</th><th>exposure levels</th></tr>
<tr><td></td><td></td><td></td><td></td><td></td><td></td><td></td><td></td><td>measurements</td></tr>
<tr><td>Haines 2001(a)

No
(mean scores as outcome, cannot convert to OR, RR)</td><td>Cross-sectional study</td><td>Study region:
Heathrow airport, London, United Kingdom

Sample population:
Children in state primary schools around Heathrow Airport in West London

Sample size:

Overall
M+F=340
M=170, F=170

Age:
mean:
9years, 8 month
range:
8years, 7month-10years,10month

Exposed/unexposed</td><td>Depression:
Child Depression Inventory (CDI)

Anxiety:
Revised Child Manifest Anxiety Scale (CMAS)

Psychological morbidity:
Strength and Difficulties Questionnaire (SDQ)

- Hyperactivity
- Psychosocial behavior
- Conduct Problems
- Emotional</td><td>Aircraft</td><td>$L_{eq,16h}$

from 1991 Civil Aviation Authority (92 days) contour map</td><td>Schools categorized by

Low noise (LN):
$L_{eq,\ 16h} \leq 57$ dB
vs
High noise (HN)
$L_{eq,\ 16h} > 63$ dB</td><td><u>Mental health outcome mean scores (from Table 2)</u>

<table>
<tr><th>Outcome</th><th>HN (4)</th><th>LN (4)</th><th>LN (3)</th><th>p (8)</th><th>p (7)</th></tr>
<tr><td>Depression</td><td>5.24</td><td>4.56</td><td>4.53</td><td>0.17</td><td>0.179</td></tr>
<tr><td>Anxiety</td><td>12.6</td><td>11.9</td><td>11.96</td><td>0.399</td><td>0.328</td></tr>
<tr><td>Hyperactivity</td><td>3.44</td><td>3.49</td><td>3.38</td><td>0.871</td><td>0.764</td></tr>
<tr><td>Psychosocial behaviour</td><td>8.22</td><td>8.11</td><td>8.02</td><td>0.65</td><td>0.314</td></tr>
<tr><td>Emot</td><td>1.95</td><td>2.13</td><td>2.02</td><td>0.49</td><td>0.971</td></tr>
</table></td><td>Study quality:
-
Cross-sectional study design

Conflict of interest:
not mentioned

Funding:
stated (local authorities and health agencies around Heathrow airport) → public funding

Confounding (matching for):
household deprivation (Townsend´s Scale: income, crowding, home ownership, unemployment), age</td></tr>
</table>

Reference (First author, publication year)<br><br>**Meta-analysis** (Yes/No)	**Study design**	**Population**	**Outcome**	**Exposure**			**Results**	**Comments**<br>(study quality [overall assessment according to SIGN/CASP], conflict of interest [stated vs. not stated], funding [financed from public funds vs. financed from industry], confounding, strengths / weaknesses [potential bias, over- or underestimation of potential effects])
		**Study region**<br>**Sample population**<br>**Sample size** (M, F, M+F):<br>**Age** (mean, range)<br>**No. of cases / no. of controls or exposed/unexposed**<br>**Time of recruitment / follow-up** (mean, range)<br>**Response (%)**<br>(baseline minus loss to follow-up)	**Disease (ICD-10)**<br>**Prescription**<br>**Questionnaire**	**exposure source**	**exposure assessment**	**exposure levels**		
		Four public schools in high aircraft noise areas (HN)<br>M+F=169, M=83, F=86<br><br>Four matched control public schools (one excluded later) in low aircraft areas (LN)<br>M+F=171, M=85, F=86<br><br>One school excluded later – biased sample (M+F=26)<br><br>**Time of recruitment/ follow-up**<br>1996<br>(testings: 3 days, each a week apart<br><br>**Response:**<br>children: 77 %<br>parents: 84%<br>teachers: 100%	symptoms<br>- Peer problems<br>- Total score<br><br>Completed at home by parents				ional symptoms — — — 1 —<br>Conduct problems: 1.5 / 1.42 / 1.27 / 0.769 / 0.246<br>Peer problems: 1.89 / 1.82 / 1.68 / 0.73 / 0.238<br>SDQ total: 8.77 / 8.86 / 8.33 / 0.902 / 0.45<br><br>Adjusted for age, deprivation and main language spoken in the four HN schools, the four LN schools and the three LN schools (excluding the procedural error school)	main language spoken at home<br><br>**Strengths/ weaknesses:**<br>+high noise selected schools were matched with low noise control schools (matched for age of the children, sound level from non-aircraft noise, noise protection in the schools, socio-economic group distribution and unemployment rate, ethnic group)<br>+ high response (and did not differ between HN and LN)<br>+ Study introduced as a Health and Environment Study →

Reference (First author, publication year)<br><br>**Meta-analysis** (Yes/No)	Study design	Population	Outcome	Exposure			Results	Comments (study quality [overall assessment according to SIGN/CASP], conflict of interest [stated vs. not stated], funding [financed from public funds vs. financed from industry], confounding, strengths / weaknesses [potential bias, over- or underestimation of potential effects])
		**Study region**<br>**Sample population**<br>**Sample size** (M, F, M+F):<br>**Age** (mean, range)<br>**No. of cases / no. of controls or exposed/unexposed**<br>**Time of recruitment / follow-up** (mean, range)<br>**Response (%)**<br>(baseline minus loss to follow-up)	**Disease (ICD-10)**<br>**Prescription**<br>**Questionnaire**	**exposure source**	**exposure assessment**	**exposure levels**		
								did not focus on noise to avoid response bias<br>+appropriate noise measurement<br>- no information on recruitment process<br>-cross-sectional design<br>- post hoc exclusion of one of the four control schools (since there were classes with lower ability rather than the requested representative children) → but results provided for both situations (without and with exclusion of the school)<br>- small difference in exposure between chronic noise exposure in low and high noise

**Reference** (First author, publication year)<br><br>**Meta-analysis** (Yes/No)	**Study design**	**Population**	**Outcome**	**Exposure**			**Results**	**Comments** (study quality [overall assessment according to SIGN/CASP], conflict of interest [stated vs. not stated], funding [financed from public funds vs. financed from industry], confounding, strengths / weaknesses [potential bias, over- or underestimation of potential effects])
		**Study region**<br>**Sample population**<br>**Sample size** (M, F, M+F):<br>**Age** (mean, range)<br>**No. of cases / no. of controls or exposed/unexposed**<br>**Time of recruitment / follow-up** (mean, range)<br>**Response (%)**<br>(baseline minus loss to follow-up)	**Disease (ICD-10)**<br>**Prescription**<br>**Questionnaire**	**exposure source**	**exposure assessment**	**exposure levels**		
								areas<br>- no other noise exposure considered<br>- air particulate level not considered
**Haines 2001**(b)<br><br>**No**<br>(mean scores as outcome, cannot convert to OR, RR)<br><br>(together with Haines 2001(a))	Longitudinal study	**Study region:**<br>Heathrow airport, London, United Kingdom<br><br>**Sample population:**<br>Children in state primary schools around Heathrow Airport in West London<br><br>**Sample size**:<br>Baseline:<br>M+F=340<br><br>Follow up:<br>M+F= 275<br>M= 132, F=143	**Depression:**<br>Child Depression Inventory (CDI)<br><br>**Anxiety:**<br>Revised Child Manifest Anxiety Scale (CMAS)	Aircraft	$L_{eq,16h}$ outdoor<br><br>from 1991 Civil Aviation Authority Leq 16h (92 days) contour map	HN schools:<br>$L_{eq,16h} \geq 66$ dB<br><br>LN schools:<br>$L_{eq,16h} \leq 57$ dB	<u>Difference in mental health scores at follow-up (low noise mean minus high-noise mean), HN=4 schools, LN=3 schools (from Table 2)</u><br><br>Outcome / HN / LN / Difference score (95% CI) / p<br>Depression (CDI) / 4.50 / 4.58 / 0.08 (-1.27, 1.42) / 0.92<br>Anxiety (CMAS) / 10.94 / 11.12 / 0.18 (-2.05, / 0.88	**Study quality:**<br>+<br>Longitudinal study design<br><br>**Conflict of interest:**<br>not mentioned<br><br>**Funding:**<br>local authorities and health agencies around Heathrow airport) → public funding<br><br>**Confounding (adjusted for):**<br>household deprivation

Reference (First author, publication year) **Meta-analysis** (Yes/No)	Study design	Population	Outcome	Exposure			Results	Comments (study quality [overall assessment according to SIGN/CASP], conflict of interest [stated vs. not stated], funding [financed from public funds vs. financed from industry], confounding, strengths / weaknesses [potential bias, over- or underestimation of potential effects])
		**Study region** **Sample population** **Sample size** (M, F, M+F): **Age** (mean, range) **No. of cases / no. of controls or exposed/unexposed** **Time of recruitment / follow-up** (mean, range) **Response (%)** (baseline minus loss to follow-up)	**Disease (ICD-10)** **Prescription** **Questionnaire**	**exposure source**	**exposure assessment**	**exposure levels**		
		**Age**<br>mean: 10 years, 8 months<br><br>**Exposed/unexposed**<br>Four public schools (1 excluded later) in high aircraft noise areas (HN)<br>N=148 (M:74, F:74)<br><br>Initially four (one excluded later→ <u>three</u> left) matched control public schools in low aircraft areas (LN)<br>M+F=127, M=: 58, F=69<br><br>**Time of recruitment / follow-up**:<br>Baseline: 1996, Follow-up: 1997<br><br>**Response**:<br>Response at baseline: NA<br>Loss to follow-up: 19%					2.38)<br><br>Adjusted for age, deprivation and main language spoken	(Townsend´s Scale), age, main language spoken at home<br><br>**Strengths/ weaknesses:**<br>+high noise selected schools were matched with low noise control schools (matching for age, sound level from non-aircraft noise, noise protection, socio-economic group, ethnic group)<br>+ longitudinal study<br>+ acceptable time for follow up (1 year)<br>+ high response (see Haines-study (945) and low lost to follow-up<br>+ appropriate noise measurement (Leq)

Reference (First author, publication year)<br><br>**Meta-analysis** (Yes/No)	**Study design**	**Population**<br>**Study region**<br>**Sample population**<br>**Sample size** (M, F, M+F):<br>**Age** (mean, range)<br>**No. of cases / no. of controls or exposed/unexposed**<br>**Time of recruitment / follow-up** (mean, range)<br>**Response (%)**<br>(baseline minus loss to follow-up)	**Outcome**<br>**Disease (ICD-10)**<br>**Prescription**<br>**Questionnaire**	**Exposure**<br>**exposure source**	**exposure assessment**	**exposure levels**	**Results**	**Comments**<br>(study quality [overall assessment according to SIGN/CASP], conflict of interest [stated vs. not stated], funding [financed from public funds vs. financed from industry], confounding, strengths / weaknesses [potential bias, over- or underestimation of potential effects])
								- small difference in exposure between chronic noise exposure in low and high noise areas<br>- no other noise exposure considered<br>- air particulate level not considered
**Haines 2001(c)**<br><br>**No**<br>(mean scores as outcome, cannot convert to OR, RR)<br><br>(togethe	Cross-sectional study	**Study region:**<br>Heathrow airport, London, United Kingdom<br><br>**Sample population:**<br>Fourth-grade pupils in schools around Heathrow Airport in West London<br><br>**Sample size:**<br>M+F= 451<br>M=229, F=222	**Psychological morbidity:**<br>Strength and Difficulties Questionnaire (SDQ)<br><br>- Hyperactivity<br>- Prosocial behavior<br>- Conduct Problems<br>- Emotional	Aircraft	$L_{eq,16h}$ outdoor<br><br>1997 Civil Aviation Authority contour map (92 days)	HN schools: $L_{eq,16h}$ ≥ 66 dB<br><br>LN schools: $L_{eq,16h}$ ≤ 57 dB	<u>Mental health outcome scores for 10 high noise schools and 10 low noise scores (from Table 3)</u><br><br>Outcome (Model 1, 2) | HN (mean, (SE) | LN (mean, (SE) | Difference score= LN-HN (95% CI) | P-value<br>hyperactivi | 4.81 (0.14) | 4.14 (0.14) | -0.66 (-1.07, | **0.0001**	**Study quality:**<br>-<br>Cross sectional study design<br><br>**Conflict of interest:**<br>not mentioned<br><br>**Funding:**<br>Department of Health and Department of Environment and Transport and the

Reference (First author, publication year)<br><br>**Meta-analysis** (Yes/No)	Study design	Population<br>**Study region**<br>**Sample population**<br>**Sample size** (M, F, M+F):<br>**Age** (mean, range)<br>**No. of cases / no. of controls or exposed/unexposed**<br>**Time of recruitment / follow-up** (mean, range)<br>**Response (%)**<br>(baseline minus loss to follow-up)	Outcome<br>**Disease (ICD-10)**<br>**Prescription**<br>**Questionnaire**	Exposure<br>**exposure source**	**exposure assessment**	**exposure levels**	Results	Comments<br>(study quality [overall assessment according to SIGN/CASP], conflict of interest [stated vs. not stated], funding [financed from public funds vs. financed from industry], confounding, strengths / weaknesses [potential bias, over- or underestimation of potential effects])
r with Haines 2001(a & b))		**Age**:<br>mean:<br>8 years, 8 month<br>range:<br>8 years,1 month-<br>9 years,8 months<br><br>**Exposed/unexposed**<br>Ten schools in high aircraft noise areas (HN)<br>M+F=236, M=117, F=119<br><br>Ten matched control schools in low aircraft areas (LN)<br>M+F=215, M=112, F=103)<br><br>**Time of recruitment / follow-up:**<br>1997<br><br>**Response:**<br>82 % (HN= 83%, LN= 81%)	symptoms<br>- Peer problems<br>- Total score (hyperactivity, emotional, conduct and peer problems)				ty -0.262)<br>4.80 (0.14) 4.15 (0.14) -0.65 (-1.06, -0.25) **0.0001**<br>Conduct problems 1.99 (0.14) 1.81 (0.14) -0.19 (-0.56, 0.19) 0.33<br>1.20 (0.13) 1.80 (0.13) -0.19 (-0.56, 0.18) 0.30<br>Peer problems 2.15 (0.14) 2.01 (0.14) -0.14 (-0.53, 0.25) 0.48<br>2.13 (0.14) 2.03 (0.14) -0.11 (-0.49, 0.27) 0.58<br>Emotional symp 2.61 (0.16) 2.43 (0.16) -0.18 (-0.63, 0.27) 0.43	Region→ public funding<br><br>**Confounding (adjusted for):**<br>Model 1: age<br>Model 2: age, main language spoken at home, household deprivation (Townsend´s Scale)<br><br>**Strengths/ weaknesses:**<br>+high noise selected schools were matched with low noise control schools (matching for age, sex, sound level from non-aircraft noise, noise protection in schools, socio-economic group, language spoken at

Reference (First author, publication year)<br><br>Meta-analysis (Yes/No)	Study design	Population	Outcome	Exposure			Results	Comments
		**Study region**<br>**Sample population**<br>**Sample size** (M, F, M+F):<br>**Age** (mean, range)<br>**No. of cases / no. of controls or exposed/unexposed**<br>**Time of recruitment / follow-up** (mean, range)<br>**Response (%)**<br>(baseline minus loss to follow-up)	**Disease (ICD-10)**<br>**Prescription**<br>**Questionnaire**	**exposure source**	**exposure assessment**	**exposure levels**		(study quality [overall assessment according to SIGN/CASP], conflict of interest [stated vs. not stated], funding [financed from public funds vs. financed from industry], confounding, strengths / weaknesses [potential bias, over- or underestimation of potential effects])
							toms 2.58 (0.16) 2.46 (0.16) -0.13 (-0.57, 0.32) 0.58<br>SDQ total 11.56 (0.42) 10.39 (0.42) -1.17 (-2.32, -0.08) **0.04** *<br>11.51 (0.40) 10.43 (0.40) -1.08 (-2.20, 0.04) 0.06<br>Model 1: adjusted for age<br>Model 2: adjusted for age, main language spoken at home, household deprivation	school)<br>+ multilevel modelling<br>+ high response (and did not differ between HN and LN)<br>+ appropriate noise measurement<br>- no information on recruitment process<br>- only little differences between the chronic exposure in low and high noise areas<br>- no other noise exposure considered<br>- air particulate level not considered
**Hjortebjerg 2016**<br><br>**Yes**	Longitudinal study	**Study region:** Denmark<br><br>**Sample population:**<br>Children from Danish National Birth Cohort	**Psychological morbidity:**<br>Strength and Difficulties Questionnaire	Road traffic and railway traffic	**Road traffic:**<br>L_{DEN}<br>L_N (22-07h)<br><br><40 dB were set	As categorical values<br>>50 dB<br>50-55 dB	Monotonic increase in OR (per 10dB(A)) until 60-65dB for abnormal total difficulties scores and abnormal hyperactivity/inattention subscales	**Study quality:**<br>+ (to ++)<br><br>**Conflict of interest:**<br>stated

Reference (First author, publication year)<br><br>**Meta-analysis** (Yes/No)	**Study design**	**Population**	**Outcome**	**Exposure**			**Results**	**Comments**<br>(study quality [overall assessment according to SIGN/CASP], conflict of interest [stated vs. not stated], funding [financed from public funds vs. financed from industry], confounding, strengths / weaknesses [potential bias, over- or underestimation of potential effects])
		**Study region**<br>**Sample population**<br>**Sample size** (M, F, M+F):<br>**Age** (mean, range)<br>**No. of cases / no. of controls or exposed/unexposed**<br>**Time of recruitment / follow-up** (mean, range)<br>**Response (%)**<br>(baseline minus loss to follow-up)	**Disease (ICD-10)**<br>**Prescription**<br>**Questionnaire**	**exposure source**	**exposure assessment**	**exposure levels**		
		**Sample size**:<br>M+F= 46,940<br><br>**Age:**<br>7 years<br><br>**Exposed/unexposed:**<br>NA<br><br>**Time of recruitment/ follow-up:**<br>1996-2002 (pregnancy)/ follow-up when child was 7 years<br><br>**Response:**<br>no information (Jacobsen, T. N., Nohr, E. A., & Frydenberg, M. (2010). Selection by socioeconomic factors into the Danish National Birth Cohort. *European Journal of Epidemiology, 25*(5), 349-355.: response 31%)	(SDQ)<br><br>- Hyperactivity/ inattention<br>- Conduct Problems<br>- Emotional symptoms<br>- Peer problems<br>- Total score (hyperactivity, emotional, conduct and peer problems)<br><br>Divided into categories normal, borderline or abnormal (Niclasen et al 2012, Youth*in*Mind 2015)		to 40 dB<br><br>**Railway noise**<br>- L_{DEN}<br><br>< 20 dB was set to 0<br><br>Both rail and road traffic noise from 1995, 2000, 2005, and 2010 for all present and historical addresses with SoundPLAN<br><br>Air pollution<br>- NO_x	55-60 dB<br>60-65 dB<br>≥ 65 dB for road traffic noise<br><br>>60 dB, >60 dB for railway noise<br><br>and<br><br>Conti-nuous analysis (per 10dB) for road and railway	<u>Associations between exposure to noise (L_{DEN}, per 10-dB increase) during pregnancy and early childhood and child behavioral borderline or abnormal scores (from Table 2)</u><br><br>**Road traffic noise (L_{DEN}) during pregnancy: adjusted model**<br>SDQ-Score / OR (95% CI) / n<br>Emotional symptoms:<br>Normal / 1.00 / 40,245<br>Borderline / 1.00 (0.95,1.06) / 3,099<br>Abnormal / 0.97 (0.92,1.03) / 3,596<br>Conduct problems:<br>Normal / 1.00 / 40,374<br>Borderline / 0.99 (0.94, 1.05 / 4,045<br>Abnorm / 0.98 / 2,521	**Funding:**<br>European Research Council<br>Danish National Birth Cohort is a result of a major grant from Danish Epidemiology Science Centre.<br>Additional support: Pharmacy Foundation, Egmont Foundation, March of Dimes Birth Defects Foundation, Augustinus Foundation and Health Foundation.<br>+7-year follow-up supported from the Lundbeck Foundation and the Danish Medical Research Council.

Reference (First author, publication year) **Meta-analysis** (Yes/No)	**Study design**	**Population**	**Outcome**	**Exposure**			**Results**	**Comments** (study quality [overall assessment according to SIGN/CASP], conflict of interest [stated vs. not stated], funding [financed from public funds vs. financed from industry], confounding, strengths / weaknesses [potential bias, over- or underestimation of potential effects])
		**Study region** **Sample population** **Sample size** (M, F, M+F): **Age** (mean, range) **No. of cases / no. of controls or exposed/unexposed** **Time of recruitment / follow-up** (mean, range) **Response (%)** (baseline minus loss to follow-up)	**Disease (ICD-10)** **Prescription** **Questionnaire**	**exposure source**	**exposure assessment**	**exposure levels**		
							al (0.92, 1.05)<br>Hyperactivity/inattention — Normal — 1.00 — 37,799<br>Borderline — 1.01 (0.96, 1.05) — 6,097<br>Abnormal — 1.01 (0.96, 1.08) — 3,044<br>Peer relationship problems — Normal — 1.00 — 37,690<br>Borderline — 1.01 (0.97, 1.06) — 5,243<br>Abnormal — 0.99 (0.94, 1.04) — 4,007<br><br>**Road traffic noise (L_{DEN}) from birth to 7 years of age**<br>SDQ — SDQ-Score — OR (95% CI) — n	**Confounding (adjusted for):** sex, age at SDQ, gestational age, birth weight, maternal age at delivery, parity, educational level, disposable income, smoking and alcohol consumption during 1st trimester, railway and airport noise at birth (for exposure during pregnancy) and at 7 years of age, and self-reported maternal mental health problems during 1st trimester (yes/no). NO_x<br><br>**strengths/ weaknesses:** + longitudinal study

Reference (First author, publication year)<br><br>**Meta-analysis** (Yes/No)	**Study design**	**Population**	**Outcome**	**Exposure**			**Results**				**Comments**<br>(study quality [overall assessment according to SIGN/CASP], conflict of interest [stated vs. not stated], funding [financed from public funds vs. financed from industry], confounding, strengths / weaknesses [potential bias, over- or underestimation of potential effects])
		**Study region**<br>**Sample population**<br>**Sample size** (M, F, M+F):<br>**Age** (mean, range)<br>**No. of cases / no. of controls or exposed/unexposed**<br>**Time of recruitment / follow-up** (mean, range)<br>**Response (%)**<br>(baseline minus loss to follow-up)	**Disease (ICD-10)**<br>**Prescription**<br>**Questionnaire**	**exposure source**	**exposure assessment**	**exposure levels**					
							Emotional symptoms	Normal	1.00	40,245	+ adequate control for confounding (including adjustment for NOx in a sensitivity analysis)<br>+ number of participants<br>+ appropriate noise exposure measurement<br>+ two noise exposures taken into account
								Borderline	1.03 (0.96, 1.10)	3,099	
								Abnormal	0.98 (0.92, 1.05)	3,596	
							Conduct problems	Normal	1.00	40,374	
								Borderline	1.01 (0.96, 1.07)	4,045	
								Abnormal	1.05 (0.98, 1.14)	2,521	
							Hyperactivity/ inattention	Normal	1.00	37,799	
								Borderline	**1.05 (1.00, 1.10)**	6,097	
								Abnormal	**1.10 (1.03, 1.18)**	3,044	

<table>
<tr>
<th rowspan="2">Reference
(First author, publication year)

Meta-analysis
(Yes/No)</th>
<th rowspan="2">Study design</th>
<th>Population</th>
<th>Outcome</th>
<th colspan="3">Exposure</th>
<th rowspan="2">Results</th>
<th rowspan="2">Comments
(study quality [overall assessment according to SIGN/CASP], conflict of interest [stated vs. not stated], funding [financed from public funds vs. financed from industry], confounding, strengths / weaknesses [potential bias, over- or underestimation of potential effects])</th>
</tr>
<tr>
<th>Study region
Sample population
Sample size (M, F, M+F):
Age (mean, range)
No. of cases / no. of controls or exposed/unexposed
Time of recruitment / follow-up (mean, range)
Response (%)
(baseline minus loss to follow-up)</th>
<th>Disease (ICD-10)
Prescription
Questionnaire</th>
<th>exposure source</th>
<th>exposure assessment</th>
<th>exposure levels</th>
</tr>
<tr>
<td></td>
<td></td>
<td></td>
<td></td>
<td></td>
<td></td>
<td></td>
<td>
<table>
<tr><td>Peer relationship problems</td><td>Normal</td><td>1.00</td><td>37,690</td></tr>
<tr><td></td><td>Borderline</td><td>1.05
(0.99, 1.10)</td><td>5,243</td></tr>
<tr><td></td><td>Abnormal</td><td>1.06
(0.99, 1.12)</td><td>4,007</td></tr>
</table>

<u>Associations between exposure to railway noise at time of birth and at SDQ (7 years), and abnormal scores on the total difficulties score and subscales (Table 3)</u>

Railway noise (L_{DEN}) at time of birth and abnormal scores
<table>
<tr><td>SDQ subscale</td><td>Noise exposure (dB)</td><td>OR (95% CI)</td><td>n</td></tr>
<tr><td>Emotional symptoms</td><td>Unexposed</td><td>1.00</td><td>2,957</td></tr>
<tr><td></td><td>≤ 60</td><td>1.11
(1.00, 1.23)</td><td>509</td></tr>
</table>
</td>
<td></td>
</tr>
</table>

**Reference** (First author, publication year) **Meta-analysis** (Yes/No)	**Study design**	**Population**	**Outcome**	**Exposure**			**Results**				**Comments** (study quality [overall assessment according to SIGN/CASP], conflict of interest [stated vs. not stated], funding [financed from public funds vs. financed from industry], confounding, strengths / weaknesses [potential bias, over- or underestimation of potential effects])
		**Study region** **Sample population** **Sample size** (M, F, M+F): **Age** (mean, range) **No. of cases / no. of controls or exposed/unexposed** **Time of recruitment / follow-up** (mean, range) **Response (%)** (baseline minus loss to follow-up)	**Disease (ICD-10)** **Prescription** **Questionnaire**	**exposure source**	**exposure assessment**	**exposure levels**					
								> 60	1.01 (0.83, 1.22)	130	
								Per 10	1.02 (0.94, 1.11)	639	
							Conduct problems	Unexposed	1.00	2,128	
								≤ 60	0.98 (0.87, 1.11)	313	
								> 60	0.90 (0.71, 1.13)	80	
								Per 10	0.94 (0.85, 1.04)	393	
							Hyperactivity/ inattention	Unexposed	1.00	2,570	
								≤ 60	0.94 (0.86, 1.05)	368	
								> 60	0.97 (0.79, 1.19)	106	
								Per 10	0.98 (0.87, 1..07)	474	

Reference (First author, publication year)<br><br>**Meta-analysis** (Yes/No)	**Study design**	**Population**<br>**Study region**<br>**Sample population**<br>**Sample size** (M, F, M+F):<br>**Age** (mean, range)<br>**No. of cases / no. of controls or exposed/unexposed**<br>**Time of recruitment / follow-up** (mean, range)<br>**Response (%)**<br>(baseline minus loss to follow-up)	**Outcome**<br>**Disease (ICD-10)**<br>**Prescription**<br>**Questionnaire**	**Exposure**<br>**exposure source**	**exposure assessment**	**exposure levels**	**Results**	**Comments**<br>(study quality [overall assessment according to SIGN/CASP], conflict of interest [stated vs. not stated], funding [financed from public funds vs. financed from industry], confounding, strengths / weaknesses [potential bias, over- or underestimation of potential effects])
							Peer relationship problems – Unexposed – 1.00 – 3,362<br>≤ 60 – 0.98 (0.89, 1.09) – 509<br>> 60 – 0.97 (0.80, 1.16) – 136<br>Per 10 – 0.98 (0.90, 1.06) – 645<br><br>**B) Railway noise <u>(L_{DEN}) at 7 years and abnormal scores</u>**<br>SDQ – Noise exposure (dB) – OR (95% CI) – n<br>Emotional symptoms – Unexposed – 1.00 – 3,085<br>≤ 60 – 1.05 (0.94, 1.16) – 439<br>> 60 – 1.10 (0.89, 1.41) – 72	

Reference (First author, publication year)<br><br>**Meta-analysis** (Yes/No)	Study design	Population<br>**Study region**<br>**Sample population**<br>**Sample size** (M, F, M+F):<br>**Age** (mean, range)<br>**No. of cases / no. of controls or exposed/unexposed**<br>**Time of recruitment / follow-up** (mean, range)<br>**Response (%)**<br>(baseline minus loss to follow-up)	Outcome<br>**Disease (ICD-10)**<br>**Prescription**<br>**Questionnaire**	Exposure<br>**exposure source**	**exposure assessment**	**exposure levels**	Results				Comments<br>(study quality [overall assessment according to SIGN/CASP], conflict of interest [stated vs. not stated], funding [financed from public funds vs. financed from industry], confounding, strengths / weaknesses [potential bias, over- or underestimation of potential effects])
								Per 10	1.00 (0.90, 1.11)	511	
							Conduct problems	Unexposed	1.00	2,174	
								≤ 60	1.05 (0.85, 1.07)	300	
								> 60	1.01 (0.75, 1.37)	47	
								Per 10	0.95 (0.84, 1.07)	347	
							Hyperactivity/ inattention	Unexposed	1.00	2,643	
								≤ 60	0.94 (0.85, 1.07)	341	
								> 60	1.05 (0.80, 1.38)	60	
								Per 10	1.09 (0.97, 1.22)	401	
							Peer relationship	Unexposed	1.00	3,470	

Reference (First author, publication year) **Meta-analysis** (Yes/No)	**Study design**	**Population**	**Outcome**	**Exposure**			**Results**	**Comments** (study quality [overall assessment according to SIGN/CASP], conflict of interest [stated vs. not stated], funding [financed from public funds vs. financed from industry], confounding, strengths / weaknesses [potential bias, over- or underestimation of potential effects])
		**Study region** **Sample population** **Sample size** (M, F, M+F): **Age** (mean, range) **No. of cases / no. of controls or exposed/unexposed** **Time of recruitment / follow-up** (mean, range) **Response (%)** (baseline minus loss to follow-up)	**Disease (ICD-10)** **Prescription** **Questionnaire**	**exposure source**	**exposure assessment**	**exposure levels**		
							problems<br>≤ 60 0.96 (0.86, 1.06) 446<br>> 60 **1.27 (1.01, 1.58)** 91<br>Per 10 **1.13 (1.03, 1.25)** 537<br><br>**Adjustment for NOx small increases in estimates (results not shown).<br>***NOx exposure in itself (in models without adjustment for noise) was not associated with behavioral problems:<br><br>Modification of associations between time-weighted mean exposure to road traffic noise (L_{DEN}) from birth to 7 years of age (per 10-dB increase) and abnormal scores on total difficulties score and hyperactivity/inattention subscale by railway noise (from Table 4)	

Reference (First author, publication year)<br><br>**Meta-analysis** (Yes/No)	**Study design**	**Population**	**Outcome**	**Exposure**			**Results**	**Comments**<br>(study quality [overall assessment according to SIGN/CASP], conflict of interest [stated vs. not stated], funding [financed from public funds vs. financed from industry], confounding, strengths / weaknesses [potential bias, over- or underestimation of potential effects])
		**Study region**<br>**Sample population**<br>**Sample size** (M, F, M+F):<br>**Age** (mean, range)<br>**No. of cases / no. of controls or exposed/unexposed**<br>**Time of recruitment / follow-up** (mean, range)<br>**Response (%)**<br>(baseline minus loss to follow-up)	**Disease (ICD-10)**<br>**Prescription**<br>**Questionnaire**	**exposure source**	**exposure assessment**	**exposure levels**		
							Hyperactivity/inattention abnormal scores<br>Railway noise exposure (dB) — Abnormal cases, n — OR (95% CI)<br>Unexposed — 2,643 — **1.07 (1.00, 1.15)**<br>≤ 60 — 341 — **1.28 (1.06, 1.55)**<br>> 60 — 60 — 1.01 (0.63, 1.63)<br>p-Interaction= 0.23<br><br>Total difficulties abnormal scores<br>Railway noise exposure (dB) — Abnormal cases, n — OR (95% CI)<br>Unexposed — 3270 — 1.06 (0.99, 1.13)<br>≤ 60 — 420 — 1.28 (1.06, 1.35)<br>> 60 — 80 — 0.95 (0.62, 1.45)<br>p-interaction=0.67<br><br>Information of effect modification by sex, low birth weight, educational level, income	

Reference (First author, publication year)<br><br>Meta-analysis (Yes/No)	Study design	Population	Outcome	Exposure			Results	Comments (study quality [overall assessment according to SIGN/CASP], conflict of interest [stated vs. not stated], funding [financed from public funds vs. financed from industry], confounding, strengths / weaknesses [potential bias, over- or underestimation of potential effects])
		**Study region**<br>**Sample population**<br>**Sample size** (M, F, M+F):<br>**Age** (mean, range)<br>**No. of cases / no. of controls or exposed/unexposed**<br>**Time of recruitment / follow-up** (mean, range)<br>**Response (%)**<br>(baseline minus loss to follow-up)	**Disease (ICD-10)**<br>**Prescription**<br>**Questionnaire**	**exposure source**	**exposure assessment**	**exposure levels**		
							available (Table 4)	
**Lim 2018**<br><br>**Yes**	Cross-sectional	**Study region:**<br>Seoul and Ulsan, South Korea<br><br>**Sample size**:<br>M+F=918<br>M= 427, F= 491<br><br>**Sample population:**<br>students from four elementary schools and four middle-school schools<br><br>**Age:**<br>11.47 ± 1.54 years (range 9-14 years)<br><br>**Time of recruitment:**<br>June-August 2016<br><br>**Response:**<br>Not reported	Child Behavior Checklist<br><br>Internalizing problems: sum of Anxious/ Depressed, Withdrawn/ Depressed, and Somatic Complaints subscales<br><br>externalizing problem: sum of Rule-Breaking Behavior and Aggressive Behavior subscales	Road traffic noise (at residential addresses of each individual)<br><br>Data from 2014	L_{DN}	continuous	Internalizing problems (Table 5):<br>L_{DN}: OR = 1.02 (95% CI, 0.97-1.06)<br><br>Externalizing problems (Table 6):<br>L_{DN}: OR = 1.03 (95% CI, 0.98-1.08)<br><br>Total problems (Table 7):<br>**L_{DN}: OR = 1.08 (95% CI, 1.01- 1.15)**	**study quality:**<br>-<br>Cross-sectional analysis<br>**conflict of interest:**<br>stated (none declared)<br>**funding:**<br>stated (Korea Ministry of Environment (MOE) as The Environmental Health Action Program (grant number: 2014001350001)<br>**confounding (controlled for):**<br>sex, age, monthly income, premature birth, maternal age at birth, passive smoking, maternal illness during pregnancy (hypertension and Pre-

**Reference** (First author, publication year)<br><br>**Meta-analysis** (Yes/No)	**Study design**	**Population**	**Outcome**	**Exposure**			**Results**	**Comments** (study quality [overall assessment according to SIGN/CASP], conflict of interest [stated vs. not stated], funding [financed from public funds vs. financed from industry], confounding, strengths / weaknesses [potential bias, over- or underestimation of potential effects])
		**Study region**<br>**Sample population**<br>**Sample size** (M, F, M+F):<br>**Age** (mean, range)<br>**No. of cases / no. of controls or exposed/unexposed**<br>**Time of recruitment / follow-up** (mean, range)<br>**Response (%)**<br>(baseline minus loss to follow-up)	**Disease (ICD-10)**<br>**Prescription**<br>**Questionnaire**	**exposure source**	**exposure assessment**	**exposure levels**		
								eclampsia), mental disorders (ADHD, Tic disorder, conduct disorder)<br>**strengths, weaknesses:**<br>-Cross-sectional study, with basic information about chronology between exposition and outcome<br>- Air pollution not considered as confounder<br>- response not reported<br>- no information about selection of participants<br>+adequate control for confounders<br>+ adequate exposure assessment<br>(+) adequate outcome assessment but

Reference (First author, publication year)<br><br>Meta-analysis (Yes/No)	Study design	Population	Outcome	Exposure			Results	Comments<br>(study quality [overall assessment according to SIGN/CASP], conflict of interest [stated vs. not stated], funding [financed from public funds vs. financed from industry], confounding, strengths / weaknesses [potential bias, over- or underestimation of potential effects])
		**Study region**<br>**Sample population**<br>**Sample size** (M, F, M+F):<br>**Age** (mean, range)<br>**No. of cases / no. of controls or exposed/unexposed**<br>**Time of recruitment / follow-up** (mean, range)<br>**Response (%)**<br>(baseline minus loss to follow-up)	**Disease (ICD-10)**<br>**Prescription**<br>**Questionnaire**	**exposure source**	**exposure assessment**	**exposure levels**		
								parental reporting<br>+ approval by the Institutional Review Board of Uslan University Hospital
**Stansfeld 2009**<br><br>**No** (continuous outcome)	Cross-sectional study	**Study region:**<br>Area around Heathrow airport (London), Schiphol Airport (Amsterdam), and Barajas airport (Madrid)<br><br>**Study population:**<br>RANCH Study<br>Pupils from 89 schools<br>Schools excluded if highly sound insulated or exposed to a different dominant noise source than to aircraft or road traffic noise.<br>Schools classified on a 4x4 grid ranging from low to high for aircraft noise and low to high for	**Psychological morbidity:**<br>Strength and Difficulties Questionnaire (SDQ)<br><br>- Hyperactivity/ inattention<br>- Conduct Problems<br>- Emotional symptoms<br>- Peer problems<br>- Prosocial behavior<br>- Total score	Aircraft and road traffic noise	Noise exposure assessments at schools<br><br>Aircraft noise: $L_{eq,16h}$ (07-23h) noise contours<br><br>Road traffic noise:<br><br>In UK and Spain estimates of road traffic noise based on simplified CRTN	30-77dB for aircraft noise<br><br>32-71dB for road traffic noise<br><br>Continuous analysis	<u>Exposure to aircraft noise traffic in dB and mental health outcomes (Table 2)</u><br>B (95%CI) / p-value<br>**Overall difficulties**<br>Model 2 0.013 (-0.023, 0.010) 0.471<br>**Hyperactivity**<br>Model 2 0.013 (0.001, 0.024) 0.032<br>**Conduct disorder**<br>Model 2 -0.005 (-0.013, 0.003) 0.220<br>**Peer Problems**<br>Model 2 0.004 0.296	**Study quality**<br>-(to +)<br><br>**Conflict of interest**<br>Sponsors had no role in study design, data collection, analysis, interpretation or writing the report<br><br>**Funding**<br>Public<br>RANCH Study founded by European Community<br>UK co-founding by Department of

Reference (First author, publication year)<br><br>**Meta-analysis** (Yes/No)	**Study design**	**Population**	**Outcome**	**Exposure**			**Results**	**Comments** (study quality [overall assessment according to SIGN/CASP], conflict of interest [stated vs. not stated], funding [financed from public funds vs. financed from industry], confounding, strengths / weaknesses [potential bias, over- or underestimation of potential effects])
		**Study region**<br>**Sample population**<br>**Sample size** (M, F, M+F):<br>**Age** (mean, range)<br>**No. of cases / no. of controls or exposed/unexposed**<br>**Time of recruitment / follow-up** (mean, range)<br>**Response (%)**<br>(baseline minus loss to follow-up)	**Disease (ICD-10)**<br>**Prescription**<br>**Questionnaire**	**exposure source**	**exposure assessment**	**exposure levels**		
		road traffic noise. Two schools/country in each noise exposure grid cell<br><br>**Sample size:**<br>M+F= 2014<br>Not separated by gender<br><br>**Age:**<br>9-10 years<br><br>**Exposed/unexposed:**<br>NA<br><br>**Time of recruitment/follow-up:**<br>Not specified<br><br>**Response rate (%)**<br>Overall child response rate: 80%<br>Parental response rate: 80%	(hyperactivity, emotional, conduct and peer problems)<br><br>Parental version		noise prediction method using a combination of proximity to roads. Measurements confirmed these estimates. For Netherlands aircraft and outdoor road traffic noise measurements provided by modelled data lined to school locations by geographical information systems.		(-0.004, 0.012)<br>**Prosocial behavior**<br>Model 2 0.002 0.720<br>(-0.007, 0.010)<br>**Emotional Problems**<br>Model 2 0.001 0.785<br>(-0.009, 0.011)<br>Model 2: adjusted for age, gender, country, mother's education, employment status, crowding, homeownership, long-standing illness, main language spoken at home, parental support, classroom glazing and **other noise exposure**<br><br><u>Exposure to road noise traffic in dB and mental health outcomes</u><br>**B (95%CI)** **p-value**<br>**Overall difficulties**<br>Model 2 -0.018 0.275<br>(-0.049, 0.013)<br>**Hyperactivity**	Environment, Food and Rural Affairs.<br>Netherlands co-founding by the Dutch Ministry of Spatial Planning, Housing and Environment and the Dutch Ministry of Transport, Public Works and Water Management<br><br>**Confounding**<br>Age, gender, country, mothers education, employment status, crowding, homeownership, illness, main language spoken at home, parental support, classroom glazing

Reference (First author, publication year) **Meta-analysis** (Yes/No)	Study design	Population	Outcome	Exposure			Results	Comments (study quality [overall assessment according to SIGN/CASP], conflict of interest [stated vs. not stated], funding [financed from public funds vs. financed from industry], confounding, strengths / weaknesses [potential bias, over- or underestimation of potential effects])
		**Study region** **Sample population** **Sample size** (M, F, M+F): **Age** (mean, range) **No. of cases / no. of controls or exposed/unexposed** **Time of recruitment / follow-up** (mean, range) **Response (%)** (baseline minus loss to follow-up)	**Disease (ICD-10)** **Prescription** **Questionnaire**	**exposure source**	**exposure assessment**	**exposure levels**		
							Model 2 0.0002 (-0.014, 0.014) 0.982<br>**Conduct disorder**<br>Model 2 -0.010 (-0.020, -0.001) 0.033<br>**Peer Problems**<br>Model 2 -0.009 (-0.019, 0.001) 0.072<br>**Prosocial behavior**<br>Model 2 -0.004 (-0.014, 0.007) 0.490<br>**Emotional Problems**<br>Model 2 0.001 (-0.011, 0.014) 0.828<br><br>Model 2: adjusted for age, gender, country, mother's education, employment status, crowding, homeownership, long-standing illness, main language spoken at home, parental support, classroom glazing and other noise exposure	**Strengths/weaknesses:**<br>+ Studied both aircraft and road traffic noise<br>+ Good noise exposure assessment<br>+ Confounder list pretty inclusive, but missing air particulate level<br>+ Good response rate<br>+ all schools matched according to socio-economic status and ethnicity within each country<br>+ study introduced as study on environment and health without explicit mention of noise<br>+ multi-country<br>- Only noise exposure at school considered<br>- cross-sectional study

Reference (First author, publication year)<br><br>**Meta-analysis** (Yes/No)	Study design	Population	Outcome	Exposure			Results	Comments (study quality [overall assessment according to SIGN/CASP], conflict of interest [stated vs. not stated], funding [financed from public funds vs. financed from industry], confounding, strengths / weaknesses [potential bias, over- or underestimation of potential effects])
		**Study region**<br>**Sample population**<br>**Sample size** (M, F, M+F):<br>**Age** (mean, range)<br>**No. of cases / no. of controls or exposed/unexposed**<br>**Time of recruitment / follow-up** (mean, range)<br>**Response (%)**<br>(baseline minus loss to follow-up)	**Disease (ICD-10)**<br>**Prescription**<br>**Questionnaire**	**exposure source**	**exposure assessment**	**exposure levels**		
								- air particulate level not considered<br>- no information on the sampling method (selection)
**Tiesler 2013**<br><br>**Yes**	Cross-sectional study	**Study region:**<br>Munich, Germany<br><br>**Study population:**<br>population-based, 10 year old children from GINIplus and LISAplus cohort<br><br>**Sample size:**<br>M+F=872<br>F=410<br>M=462<br><br>**Age:**<br>10.1 +/- 0.22 years	**Psychological morbidity**<br>Strength and Difficulties Questionnaire (SDQ)<br><br>- Hyperactivity/ inattention<br>- Conduct Problems<br>- Emotional symptoms<br>- Total score (hyperactivity, emotional,	Road traffic noise	L_{DEN}<br>L_N (22-06h)<br><br>Noise modeling based on Munich noise map from 2007	L_{DEN}<br>Most exposed façade= 52.42+/- 7.87 dB range **35.40**-74.70 dB<br><br>Least exposed= 44.92+/- 6.15 dB range: 24.20-	<u>Association between road traffic noise variables (L_{DEN}) at most and least exposed façade of the children's' home address and behavioral problems (from Table 4) using continuation odds ratio (per IQR)</u><br><br>OR / 95% CI<br>L_{DEN} at most exposed façade<br>*These estimates were included in the meta-analysis and converted to OR per 10dB*<br>Total difficulties score: 1.16 / 0.95-1.40<br>Emotional symptoms: 1.14 / 0.95-1.37	**Study quality**<br>**-**<br><br>**Conflict of interest**<br>No information<br><br>**Funding**<br>Federal Ministry for Education, Science, Research and Technology, Helmholtz Zentrum München, Federal Ministry of Environment, Ludwig/Maximilians-

Reference (First author, publication year)<br><br>**Meta-analysis** (Yes/No)	**Study design**	**Population**	**Outcome**	**Exposure**			**Results**	**Comments** (study quality [overall assessment according to SIGN/CASP], conflict of interest [stated vs. not stated], funding [financed from public funds vs. financed from industry], confounding, strengths / weaknesses [potential bias, over- or underestimation of potential effects])
		**Study region**<br>**Sample population**<br>**Sample size** (M, F, M+F):<br>**Age** (mean, range)<br>**No. of cases / no. of controls or exposed/unexposed**<br>**Time of recruitment / follow-up** (mean, range)<br>**Response (%)**<br>(baseline minus loss to follow-up)	**Disease (ICD-10)**<br>**Prescription**<br>**Questionnaire**	**exposure source**	**exposure assessment**	**exposure levels**		
		**No. of cases**<br>NA<br><br>**Time of recruitment/follow-up:**<br>Baseline LISAplus:<br>Dec 1997-Jan 1999<br>Baseline GINIplus<br>Sept 1995-June 1998<br>Time of current study:<br>10-year follow-up of both cohorts<br><br>**Response rate (%)**<br>NA	conduct and peer problems)<br><br>German version, parent-reported			64.50 dB<br><br>L_N<br>Most exposed façade= 43.36+/-7.63 dB range **26.90-**65.70dB<br><br>Least exposed= 35.96+/-6.27dB range: 15.40-55.40dB<br><br>Risk change	Conduct problems 0.95 0.76-1.18<br>Hyperactivity/ inattention **1.28** **1.03-1.58**<br>Peer relationship problems 0.93 0.72-1.21<br><br>L_{DEN} at least exposed facade<br>Total difficulties score 1.16 0.91-1.46<br>Emotional symptoms - -<br>Borderline/abnormal vs normal 1.18 0.92-1.51<br>**Abnormal vs borderline** **2.19** **1.32-3.64**<br>Conduct problems 0.93 0.72-1.20<br>Hyperactivity/ 1.18 0.91-1.52	University Munich (public funding)<br><br>**Confounding**<br>Study, sex, age, parental educational level, mothers age at birth, television/computer usage, single parent status<br><br>**Strengths/ weaknesses:**<br><br>+ appropriate exposure measure with 2 noise indicators<br>- not adjusted for other confounders (i.e. air particulate level)<br>-no other noise exposure taken into account

Reference (First author, publication year) **Meta-analysis** (Yes/No)	**Study design**	**Population**	**Outcome**	**Exposure**			**Results**	**Comments** (study quality [overall assessment according to SIGN/CASP], conflict of interest [stated vs. not stated], funding [financed from public funds vs. financed from industry], confounding, strengths / weaknesses [potential bias, over- or underestimation of potential effects])
		**Study region** **Sample population** **Sample size** (M, F, M+F): **Age** (mean, range) **No. of cases / no. of controls or exposed/unexposed** **Time of recruitment / follow-up** (mean, range) **Response (%)** (baseline minus loss to follow-up)	**Disease (ICD-10)** **Prescription** **Questionnaire**	**exposure source**	**exposure assessment**	**exposure levels**		
						per IQR (equal approx. to 8.2- 9 dB)	inattention<br>Peer relationship problems 0.94 0.70-1.28<br>L_N at most exposed facade<br>Total difficulties score 1.18 0.97-1.44<br>Emotional symptoms 1.17 0.97-1.41<br>Conduct problems 0.95 0.76-1.19<br>**Hyperactivity/ inattention 1.32 1.06-1.64**<br>Peer relationship problems 0.92 0.70-1.21<br>L_N at least exposed facade<br>Total difficulties score 1.17 0.92-1.48<br>Emotional symptoms - -<br>Borderline/abnor 1.19 0.93-1.54	- cross-sectional design<br>- not known response rate

Reference (First author, publication year)<br><br>**Meta-analysis** (Yes/No)	**Study design**	**Population**<br>**Study region**<br>**Sample population**<br>**Sample size** (M, F, M+F):<br>**Age** (mean, range)<br>**No. of cases / no. of controls or exposed/unexposed**<br>**Time of recruitment / follow-up** (mean, range)<br>**Response (%)**<br>(baseline minus loss to follow-up)	**Outcome**<br>**Disease (ICD-10)**<br>**Prescription**<br>**Questionnaire**	**Exposure**<br>**exposure source**	**exposure assessment**	**exposure levels**	**Results**	**Comments**<br>(study quality [overall assessment according to SIGN/CASP], conflict of interest [stated vs. not stated], funding [financed from public funds vs. financed from industry], confounding, strengths / weaknesses [potential bias, over- or underestimation of potential effects])
							mal vs normal<br>**Abnormal vs borderline** **2.29** **1.36-3.85**<br>Conduct problems 0.93 0.72-1.20<br>Hyperactivity/ inattention 1.19 0.92-1.55<br>Peer relationship problems<br>Borderline/abnormal vs normal 1.08 0.77-1.53<br>Abnormal vs borderline 0.49 0.24-1.00<br>Adjusted by study, sex, age, parental educational level, mothers age at birth, television/computer usage, single parent status	
**Weyde 2017**	Cohort study	**Study region:**<br>Oslo, Norway	Inattention at age 8 from Rating Scale	Road traffic noise	LDEN	continuous	Table 4: Road traffic noise and inattention score (main model- ANOVA)	**study quality:**<br>+

<table>
<tr><th rowspan="2">Reference
(First author, publication year)

Meta-analysis (Yes/No)</th><th rowspan="2">Study design</th><th>Population</th><th>Outcome</th><th colspan="3">Exposure</th><th rowspan="2">Results</th><th rowspan="2">Comments
(study quality [overall assessment according to SIGN/CASP], conflict of interest [stated vs. not stated], funding [financed from public funds vs. financed from industry], confounding, strengths / weaknesses [potential bias, over- or underestimation of potential effects])</th></tr>
<tr><th>Study region
Sample population
Sample size (M, F, M+F):
Age (mean, range)
No. of cases / no. of controls or exposed/unexposed
Time of recruitment / follow-up (mean, range)
Response (%)
(baseline minus loss to follow-up)</th><th>Disease (ICD-10)
Prescription
Questionnaire</th><th>exposure source</th><th>exposure assessment</th><th>exposure levels</th></tr>
<tr><td>
No
(continuous endpoints)</td><td>
Norwegian Mother and child cohort study (MoBa)</td><td>
Sample size:
N_{Total}=14,032 MoBa participants
M+P=1,934 children (pregnancy study sample)
N2=1,384 children (postnatal study sample)

Males and females about equally distributed (%boys: 47.5-52.5 (distinction between pregnancy sample and postnatal sample and further between different exposure level))

Sample population:
Children born between 2004 and 2007

Age:
month: 97.3-97.5 (distinction between pregnancy sample and postnatal sample and further</td><td>for Disruptive Behavior
->corresponding to 9 inattention items of ADHD in DSM-IV</td><td>
At child's residence and mother's residence during pregnancy

Input data from 2006 and 2011

Assessment of railway traffic noise and NO_2, NO_x and $PM_{2.5}$ -> confounders</td><td></td><td></td><td><table><tr><th></th><th>N</th><th>coef</th><th>95%CI</th></tr><tr><td>Pregnancy</td><td>1934</td><td>0.0042</td><td>-0.0013-0.0096</td></tr><tr><td>**Postnatal**</td><td>**1384**</td><td>**0.0083**</td><td>**0.0012-0.0154**</td></tr><tr><td>**5-years average**</td><td>**1384**</td><td>**0.0090**</td><td>**0.0016-0.0164**</td></tr></table>Different sensitivity analysis performed: stratified by maternal education, household income, with railway noise, without parents living apart, without premature birth, without low birth weight, with air pollution
Sensitivity analysis for postnatal and pregnancy road traffic considering the other noise time (pregnancy, postnatal, 5-year average) as covariates in the model.-> Similar results.
Results were not extracted</td><td>Cohort study
conflict of interest:
stated (none declared)
funding:
stated (Norwegian Research Council, Environmental Exposures and Health Outcomes (MILPAAHEL), project no.; 228,142. The Norwegian Mother and Child Cohort Study is supported by the Norwegian Ministry of Health and Care Services and the Ministry of Education and Research, NIH/NIEHS (contract no N01-ES-75558), NIH/NINDS (grant no.1 UO1 NS 047537–01 and</td></tr>
</table>

**Reference** (First author, publication year)<br><br>**Meta-analysis** (Yes/No)	**Study design**	**Population**	**Outcome**	**Exposure**			**Results**	**Comments** (study quality [overall assessment according to SIGN/CASP], conflict of interest [stated vs. not stated], funding [financed from public funds vs. financed from industry], confounding, strengths / weaknesses [potential bias, over- or underestimation of potential effects])
		**Study region**<br>**Sample population**<br>**Sample size** (M, F, M+F):<br>**Age** (mean, range)<br>**No. of cases / no. of controls or exposed/unexposed**<br>**Time of recruitment / follow-up** (mean, range)<br>**Response (%)**<br>(baseline minus loss to follow-up)	**Disease (ICD-10)**<br>**Prescription**<br>**Questionnaire**	**exposure source**	**exposure assessment**	**exposure levels**		
		between different exposure level)<br>**Time of recruitment:**<br>1998-2008<br><br>**Response:**<br>40.6%<br>Participant/ non-participant analysis: slight differences between postnatal sample and non-participants: postnatal sample higher household income and higher % of mothers drinking alcohol during pregnancy						grant no.2 UO1 NS 047537-06A1))<br>**confounding (controlled for):**<br>age, gender, household income, maternal education, urbanity, ethnicity, maternal alcohol consumption during pregnancy, maternal smoking during pregnancy, low birth weight, prematurity<br>**strengths, weaknesses:**<br>- statistical analysis<br>+ participant/ non-participant analysis<br>+ adequate control for confounders<br>+ adequate exposure assessment<br>+adequate control for

**Reference** (First author, publication year) **Meta-analysis** (Yes/No)	**Study design**	**Population**	**Outcome**	**Exposure**			**Results**	**Comments** (study quality [overall assessment according to SIGN/CASP], conflict of interest [stated vs. not stated], funding [financed from public funds vs. financed from industry], confounding, strengths / weaknesses [potential bias, over- or underestimation of potential effects])
		**Study region** **Sample population** **Sample size** (M, F, M+F): **Age** (mean, range) **No. of cases / no. of controls or exposed/unexposed** **Time of recruitment / follow-up** (mean, range) **Response (%)** (baseline minus loss to follow-up)	**Disease (ICD-10)** **Prescription** **Questionnaire**	**exposure source**	**exposure assessment**	**exposure levels**		
								confounders<br>+ ethics approval by The Regional Committee for Medical Research Ethics<br>(+) adequate definition and assessment of outcome (but parental reporting)<br>(+) sensitivity analysis for air pollution

IQR Interquartile range
